# On the potential alternate binding change mechanism in a dimeric structure of Pyruvate Phosphate Dikinase

**DOI:** 10.1038/s41598-017-08521-w

**Published:** 2017-08-14

**Authors:** Daniel Ciupka, Holger Gohlke

**Affiliations:** 1 Institute of Pharmaceutical and Medicinal Chemistry, Heinrich-Heine-Universität Düsseldorf, 40225 Düsseldorf, Germany; 2 John von Neumann Institute for Computing (NIC), Jülich Supercomputing Centre (JSC) & Institute for Complex Systems - Structural Biochemistry (ICS 6), Forschungszentrum Jülich, 52425 Jülich Germany

## Abstract

The pyruvate phosphate dikinase (PPDK) reaction mechanism is characterized by a distinct spatial separation of reaction centers and large conformational changes involving an opening-closing motion of the nucleotide-binding domain (NBD) and a swiveling motion of the central domain (CD). However, why PPDK is active only in a dimeric form and to what extent an alternate binding change mechanism could underlie this fact has remained elusive. We performed unbiased molecular dynamics simulations, configurational free energy computations, and rigidity analysis to address this question. Our results support the hypothesis that PPDK dimerization influences the opening-closing motion of the NBDs, and that this influence is mediated via the CDs of both chains. Such an influence would be a prerequisite for an alternate binding change mechanism to occur. To the best of our knowledge, this is the first time that a possible explanation has been suggested as to why only dimeric PPDK is active.

## Introduction

Pyruvate phosphate dikinase (PPDK) is a key enzyme in the cellular energy metabolism that catalyzes the ATP- and phosphate (P_i_)-dependent conversion of pyruvate to phosphoenolpyruvate (PEP) in C_4_ plants and the reverse, ATP-forming reaction in bacteria and protozoa. The reaction involves two independent partial reactions in plants^1^ and protozoa^2^, with a phosphoryl transfer mediated by the catalytic histidine H456^3–5^ (amino acid numbering according to the PPDK sequence of *Flaveria trinervia*):

1 $$Enzyme\,+ATP+{P}_{i}\rightleftharpoons Enzym\mbox{--}His\mbox{--}P+AMP\,+PP$$

2 $$Enzyme\mbox{--}His\mbox{--}P+Pyruvate\rightleftharpoons Enzyme\,+PEP$$

**Figure 1:**
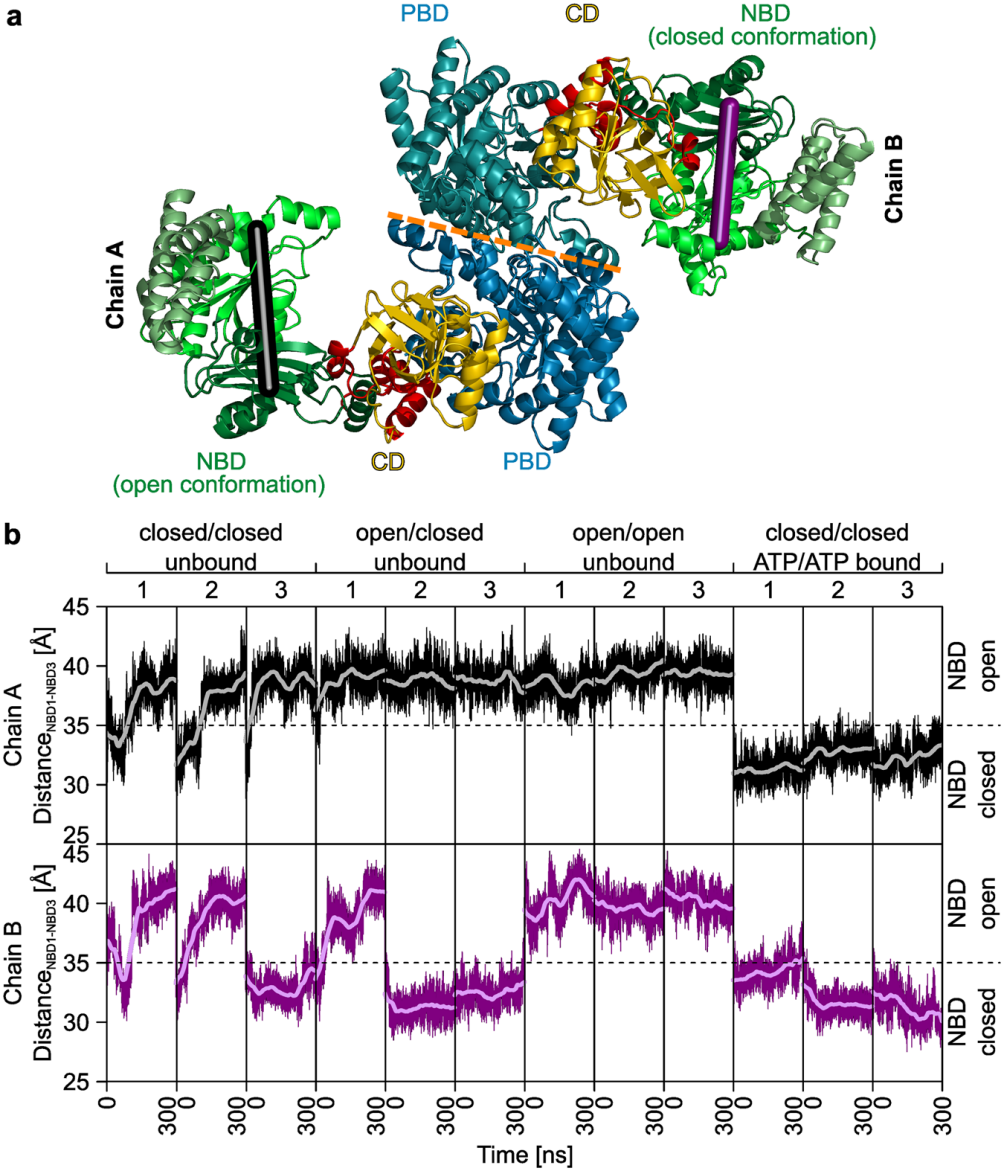
Unrestrained MD simulations of dimeric PPDK. (**a**) Crystal structure PDB ID 5JVJ showing two monomers (chains A, B) of PPDK in the asymmetric unit. The central domain (CD, yellow) contains the catalytic histidine. At the nucleotide-binding domain (NBD, with the three subdomains depicted by three different greens) and the PEP/pyruvate-binding domain (PBD, colored blue), partial reactions (1) and (2) (see Introduction section) take place. The linker domains (LD, colored red) connect CD, PBD, and NBD. The dimer interface (indicated by the dashed orange line) is formed by the PBDs of both monomers. The NBDs of chain A and B show open and closed conformations, respectively. The distance_NBD1-NBD3_ measured between S215_Cα_ – E272_Cα_ is used as reaction coordinate for the opening-closing motion of chain A (black line) and chain B (deep purple line). (**b**) Time course of the opening-closing motions of the NBDs of chain A (black line) and chain B (deep purple line) measured by the respective distance_NBD1-NBD3_. From the conformations labeled on the top axis, three replica MD simulations each were started of 300 ns length, with the mean over an interval of 60 ns shown in gray (for chain A) and light magenta (for chain B).

The temporal separation of the partial reactions is mirrored by a spatial separation of reaction centers^6, 7^: PPDK consists of three domains, an N-terminal nucleotide-binding domain (NBD), the location of partial reaction (1), a C-terminal PEP/pyruvate-binding domain (PBD), the location of partial reaction (2), and a central domain (CD), the location of H456 (Fig. 1a). To date, 13 crystal structures of PPDK have been resolved^8^, for PPDKs of the bacterium *Clostridium symbiosum* ^7, 9–12^, the protozoan *Trypanosoma brucei* ^13^, the C_4_ plants *Zea mays* ^14^ and *Flaveria trinervia* ^8^, and the C_3_ plant *Flaveria pringlei* ^8^. Structural clustering of the PPDK structures reveals two principal movements (Supplementary Fig. S1): First, the NBD, which consists of three subdomains forming the ATP-grasp motif^15^, shows an opening-closing motion assumed to be associated with ATP binding^13^. Second, the CD is either located near the PBD, between the PBD and NBD, or near the NBD. Accordingly, and in line with the enzyme mechanism, the CD has been suggested to undergo a swiveling motion to shuttle the phosphoryl group between the two active sites, followed by a backward motion to initiate the next cycle of phosphoryl transfer^7^. The swiveling motion involves a rotation of ∼110° of the CD, which transports H456 across a distance of ~45 Å^7^. This makes the swiveling motion of PPDK one of the largest single-domain movements observed in proteins yet.

Recently, we predicted by unrestrained molecular dynamics (MD) simulations and 1D and 2D configurational free energy (potential of mean force (PMF)) calculations on *monomeric* PPDK that the swiveling motion proceeds via a conformational intermediate where the CD is located roughly in between the PBD and NBD^8^. A crystal structure of PPDK from *Flaveria trinervia* confirmed this, until then, unknown intermediate (PDB ID 5JVJ)^8^. The molecular simulations and crystallographic data furthermore indicated that PPDK might employ a Brownian ratchet mechanism biasing thermal fluctuations in order to generate a net directional CD motion^8^. Finally, our calculations revealed an intramolecular coupling between the CD motion and the opening-closing motion of the NBD, which was corroborated by the comparative analysis of available PPDK crystal structures^8^. While the biologically active form of PPDK has been considered a dimer in bacteria, and may be a tetramer in several plants^16–18^, the 5JVJ structure exposed for the first time the *dimeric* form of PPDK in the asymmetric unit^8^, with the dimer interface formed by the two PBDs (Fig. 1a). Notably, the two monomers differ in the conformational state of the NBDs, with that of chain A being in an open and that of chain B in a closed conformation. The NBD of monomer B exhibits additional electron density that might reflect a bound adenine nucleotide. The distinct conformational states of the NBD of 5JVJ have led to the hypothesis that PPDK employs an alternate binding change mechanism^8^ (also termed reciprocating mechanism stressing its processivity^19^) similar to ATP synthase^20, 21^ or bacterial ATP-dependent DNA helicases^22^. However, no further detailed insights if and how such an alternate binding change mechanism occurs in a dimeric PPDK structure have yet been presented. Furthermore, it has remained elusive if such a mechanism underlies the activation of PPDK in the dimeric form.

Here, we investigate the structural dynamics of the PPDK dimer from *F. trinervia* by unrestrained all-atom MD simulations of an aggregate simulation time of 3.6 μs (Table 1), the energetics of the opening-closing motion of one NBD as a function of the open/closed state of the other NBD as well as the conformational state of the CD in the PPDK dimer by PMF calculations of in total 2.6 μs sampling time (Supplementary Table S1), and the influence of dimerization on the structural stability by rigidity theory-based Constraint Network Analysis (CNA). Our computations support the hypothesis that PPDK dimerization does influence the opening-closing motion of the NBDs, and that this influence is mediated via the CDs of both chains.

**Table 1:** MD simulations of the PPDK dimer.

Conformation chain A	Conformation chain B	Repetitions	Simulation length^[a]^
NBD closed	NBD closed	3	300
NBD open	NBD closed	3	300
NBD open	NBD open	3	300
NBD closed, ATP bound	NBD closed, ATP bound	3	300

^[a] ^In ns.

## Results and Discussion

### Native interface of the PPDK dimer

Initially, we investigated the interface found between the PBD domains in 5JVJ as to its role for forming the dimeric biological unit of *F. trinervia* PPDK. Using the PDBePISA tool^23^, the analysis revealed that this interface is the only one in this crystal structure suggested to be functionally relevant (complexation significance score (CSS): 0.541; CSS of all other interfaces: zero), with an interface area of 2077 Å^2^, > 40 potential hydrogen bonds and salt bridges and > 65 potential hydrophobic interactions formed across the interface, and a Δ*G p*-value of 0.370 (Supplementary Table S2). Furthermore, mutations reported to reduce PPDK’s cold-dependent dissociation and, therefore, inactivation^24^ are located in the vicinity of the dimer interface (Supplementary Fig. S2a), and surface residues forming the dimer interface are conserved (between 25% and 100%, Supplementary Fig. S2b and Supplementary Table S3). The only other two regions with similarly high conservations of surface residues are in the vicinities of the functionally relevant active sites of the NBD and the PBD, where the CD docks during the swiveling motion (Supplementary Fig. S2b). The high degree of sequence conservation thus correlates with the functional importance of these regions. Together, these results imply that the interface found between the PBD domains plays an essential role in complex formation rather than it being a result of crystal packing only.

### Structural dynamics of dimeric PPDK

Next, we generated all-atom structures of PPDK dimers from *F*. *trinervia* with the two NBDs in unbound closed/closed, open/closed, or open/open conformations, as well as in ATP-bound closed/closed conformations. The dimers served as starting structures for three independent MD simulations of 300 ns length each (Table 1), respectively, in total, resulting in twelve MD simulations (Fig. 1b). The setup in terms of force field and simulation parameters was identical to the one used successfully in ref. 8 on monomeric PPDK, applying, in addition, force field parameters for ATP and Mg^2+^ (see Materials and Methods for details). The MD simulations revealed a pronounced tendency for unbound, closed NBD domains to open (six out of nine cases) with an opening usually occurring during the first 150 ns of a trajectory. In contrast, open NBD conformations remained open during the simulation time (nine out of nine cases), and ATP-bound closed conformations remained closed (six out of six cases), although it cannot be excluded that this finding is due to too short simulation times, particularly in the last case. Still, with respect to the question of an alternate binding change mechanism, ten out of twelve MD simulations do not indicate a preference for one NBD of the PPDK dimer to be in a conformation different from the other, given identical binding states of both domains. Note that the CDs stayed at their starting positions facing the PBD in all MD simulations, in contrast to the observation from our study on monomeric PPDK^8^. The latter observation might result from MD simulation times that are at least two-fold longer than those here^8^.

**Figure 2:**
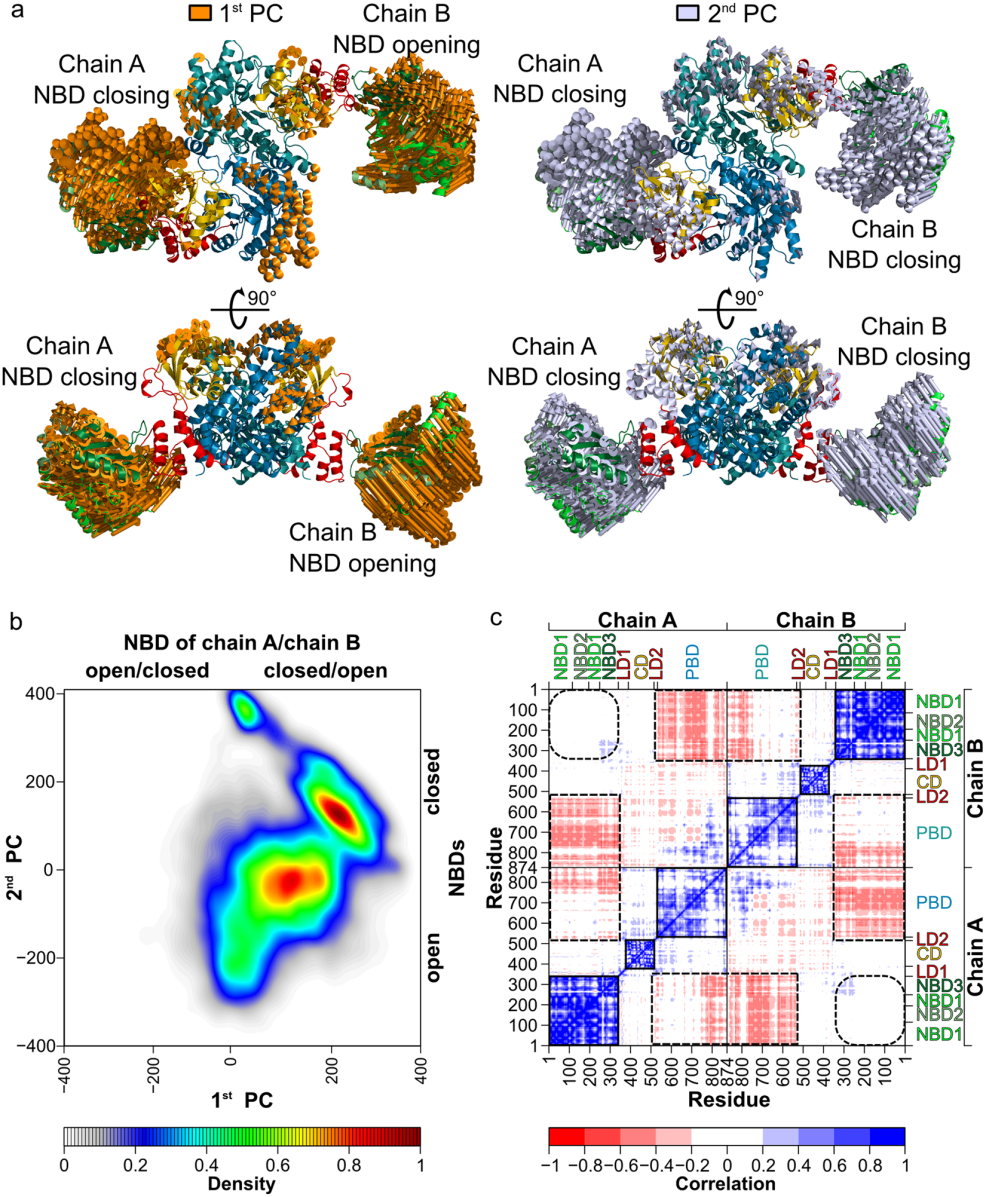
Correlation of motions in the PPDK dimer. (**a**) Representation of atomic displacements along the directions of the 1^st^ (gold, left) and 2^nd^ (silver, right) principle component, computed from MD simulations of an aggregate simulation time of 3.6 μs. The 1^st^ PC describes an antisymmetric motion of both NBDs (i.e., one opens, the other closes), the 2^nd^ describes a symmetric one (i.e., both open, or close, simultaneously). The amplitudes of the motions were scaled, and a cutoff for small displacements was applied for best graphical representation. (**b**) Density distribution of conformations from the twelve MD simulations mapped onto the plane spanned by the 1^st^ and 2^nd^ PCs. The highest density has been normalized to 1, and all other densities accordingly. **(c)** Cross-correlation map of C_α_ atom fluctuations from MD simulations with the residue numbering labeled on the left and at the bottom, and the substructures labeled on the right and top axes. Positive correlations are indicated in blue, negative correlations in red (see color scale). Correlated motions within and between domains are marked with squares with solid and dashed lines, respectively. Squares with round corners indicate that no correlated motions between the two NBDs were found.

Furthermore, a principle component analysis (PCA) in Cartesian space was performed on coordinates derived from all twelve MD simulations. 66% of the variance in the motions of the PPDK dimers can be characterized by the first two principal components (PC), with the 1^st^ and 2^nd^ PC explaining ~34%, and ~32%, respectively (Supplementary Fig. S3a). According to atomic displacements along PC directions (Fig. 2a), both the 1^st^ and 2^nd^ PC primarily reflect the opening-closing motions of both NBDs, which are predominantly executed by the first and second subdomains (NBD1 and NBD2), as indicated by (sub)domain-wise collectivity indices (Supplementary Fig. S3b). Interestingly, while the 1^st^ PC describes an antisymmetric motion of both NBDs (i.e., one opens, the other closes), the 2^nd^ describes a symmetric one (i.e., both open, or close, simultaneously) (Fig. 2a,b,c). Considering the almost equal proportion of variance both PCs characterize, this finding implies that the opening-closing motion of one NBD is not directly dependent on the opening-closing motion of the other NBD.

Moreover, cross-correlation analysis of C_α_ atom fluctuations, computed to identify a possible coupling between the opening-closing motions, reveals, first, positively correlated motions of the PPDK domains (NBDs, CDs, and PBDs) themselves, and, second, in part positively and in part weakly negatively correlated motions between the PBDs (Fig. 2b); the positively correlated motions occur in those regions of the PBDs that form the dimer interface (Supplementary Fig. S4b). Third, motions of the NBD of one chain are weakly negatively correlated to those of the PBD of the other chain, which might reflect the close distances between these domains (Fig. 1a). However, motions between the NBD of one chain and the PBD of the same chain are largely uncorrelated, as also found for monomeric PPDK^8^. Motions between both CDs are largely uncorrelated as well, as may have been expected from the CDs moving little with respect to their starting position (see above). Finally, motions between the two NBDs are also uncorrelated (mean correlation coefficient of 0.1), implying again that the opening-closing motion of one NBD is not directly dependent on the opening-closing motion of the other NBD.

### Energetics of the opening-closing motion

**Figure 3:**
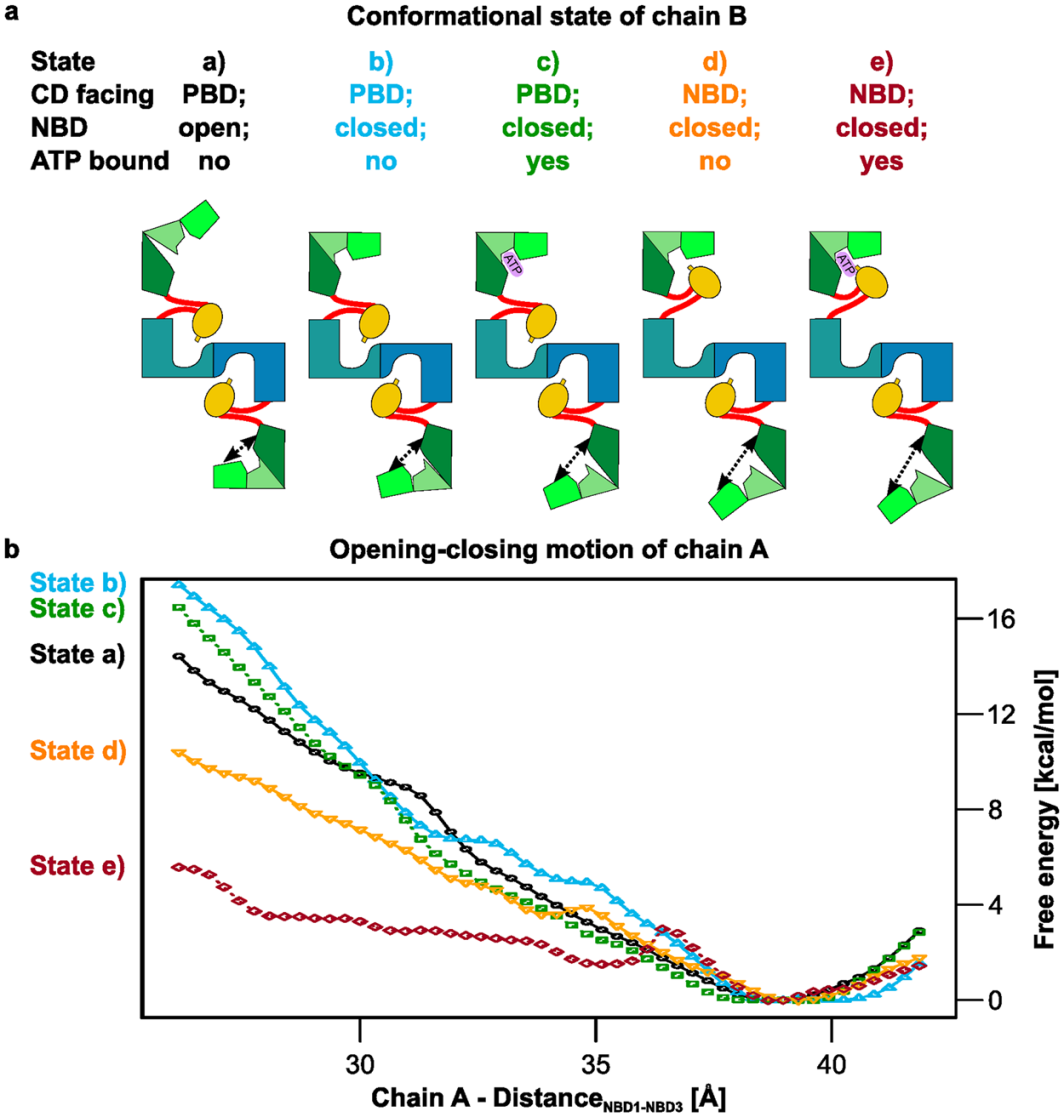
PMF of the opening-closing motion of chain A for different states of chain B. (**a**) Schematic representation of simulated systems and (**b**) PMF of the opening-closing motion of the NBD of chain A, with chain B having the CD facing the PBD with the NBD in the open, unbound conformation (a, black circle), closed, unbound conformation (b, blue triangle pointing upwards), and closed, ATP-bound conformation (c, green square), as well as the CD facing the NBD with the NBD in the closed, unbound conformation (d, orange triangle pointing downwards) and closed, ATP-bound conformation (e, red diamond). The distance_NBD1-NBD3_ between Ser215_Cα_ – Glu272_Cα_ was used as a reaction coordinate (depicted as black arrows in panel a). The standard deviation for all data points is < 0.02 kcal mol^−1^ computed by a bootstrapping procedure (see Methods section).

To complement the unrestrained MD simulations, we computed the PMF of the opening-closing motion of the NBD of chain A, using umbrella sampling along the distance_NBD1-NBD3_ between S215_Cα_ – E272_Cα_ of chain A as a reaction coordinate. This reaction coordinate had been shown to represent the opening-closing motion of the NBD very well^8^. From our previous study on the PPDK monomer^8^, we expected the open conformation of PPDK to be energetically preferred if the CD of the same chain faces the PBD. To analyze how the conformational and binding states of chain B modulate the PMF, umbrella sampling simulations were performed for five states of chain B (Fig. 3a): With the CD facing the PBD and the NBD being unbound and a) open or b) closed; c) with the CD facing the PBD and the NBD being closed and ATP-bound; with the CD facing the NBD and the NBD being closed and d) unbound or e) ATP-bound. State c) resembles what has been found in PDB ID 5JVJ. Intermediate states between the open and closed NBD conformations of chain A were generated by targeted simulations with the NMSim approach^25, 26^, as done before^8^. The umbrella windows display considerable overlap regarding the frequency distribution of values for the reaction coordinate (Supplementary Fig. S5a). Furthermore, although the motions of both CDs, the NBD of chain B, and ATP were not restrained, their conformational and binding states remain similar to the starting conditions (Supplementary Fig. S5a–d). Finally, the PMFs using ½ or all sampled data differ by at most ~2 kcal mol^−1^ (Supplementary Fig. S5e), suggesting converged result. For all systems, the global minimum is found at distance_NBD1-NBD3_ ~39 Å, demonstrating that the unbound NBD with the CD facing the PBD prefers the open conformation, as also indicated by the unrestrained MD simulations of the PPDK dimer. This result is also in line with PMF computations for the non-phosphorylated PPDK monomer^8^, the respective distance_NBD1-NBD3_ of all currently available X-ray structures with an unbound NBD and the CD facing the PBD (PDB ID: 2R82 (distance_NBD1-NBD3_ = 38.7 Å), 5JVJ (38.5 Å), 1VBG (35.8 Å), and 1VBH (36.8 Å)^8^), and the suggested catalytic mechanism^7^. Notably, the PMFs are qualitatively indistinguishable irrespective of whether chain B is in state a), b), or c) (Fig. 3), corroborating the above implications (Figs 1 and 2) that the opening-closing motion of one NBD does not directly depend on the conformational and binding state of the NBD of the other chain.

### Changes in structural stability due to PPDK dimer formation

As an independent way to analyze mechanical coupling within the PPDK dimer structure, we performed CNA^27^ on coordinates of chain A, chain B, or the dimer, which were all extracted from one MD simulation of the PPDK dimer with a conformation as observed in the X-ray structure (PDB ID 5JVJ) (see Materials and Methods for details). In CNA, the molecular system is represented as a constraint network, which is analyzed using rigidity theory^28, 29^, revealing rigid clusters and flexible links in between. By performing a constraint dilution simulation^30^, a stability map^31^ is obtained that reports on the hierarchy of structural stability of the molecular system. It does so in terms of the energy along a constraint dilution trajectory at which a rigid contact *rc*_*ij*_ between a pair of amino acids (*i*, *j*) vanishes; a rigid contact is present as long as the two residues belong to the same rigid cluster (Supplementary Fig. S6). The difference stability map calculated as *rc*_*ij*_(Dimer) – *rc*_*ij*_(Monomer, chain A) – *rc*_*ij*_(Monomer, chain B) then reports on the influence on structural stability due to dimerization. Here, only additional interactions across the interface affect the structural stability but not conformational changes, as the conformations of the dimer and the two monomer chains were extracted from one MD trajectory. Note that this analysis allows revealing long-range aspects to rigidity percolation, that is, whether a region is flexible or rigid may depend on structural details that are faraway^32^.

**Figure 4:**
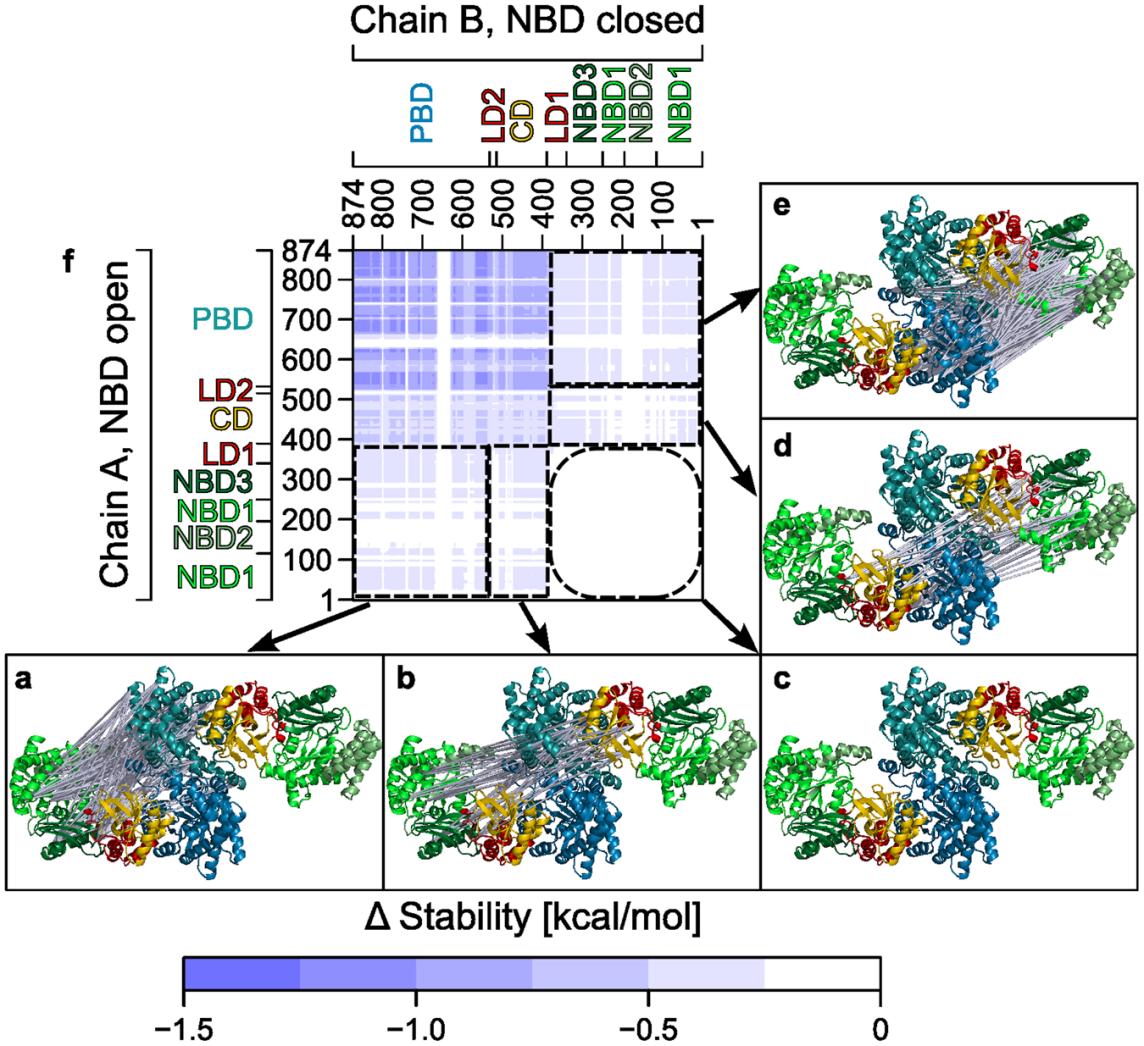
Effect of the dimerization on the stability of PPDK as computed by CNA. Difference in the stability of rigid contacts between two residues projected onto C_α_ atoms of the PPDK structure 5JVJ (missing atoms were added by homology modeling) and color-coded according to the scale at the bottom: Between (**a**) NBD and PBD, (**b**) NBD and CD, **(c)** NBD and NBD, (**d**) CD and NBD, and **(e)** PBD and NBD of chain A and chain B, respectively. (**f**) Stability map of the intermolecular differences in rigid contacts. Rigid contacts that are more stable in the dimer than in the monomer are indicated in darker blue colors. The residue numbering is given on the left and on the bottom, as are labels for the domains. For clarity rigid contacts between only every 20^th^ residue are displayed in panels (**a**) to (**e**). See Supplementary Fig. S7 for a display of all rigid contacts.

The intermolecular part of the difference stability map was projected onto the PPDK dimer structure in terms of struts connecting respective residue pairs; the color of the struts indicates to what extent a rigid contact was stabilized due to formation of the PPDK dimer from the monomers (Fig. 4 and Supplementary Fig. S7). Dimerization does not only increase structural stability of both PBDs, as may have been expected because of additional interactions across the interface, (Fig. 4f and Supplementary Fig. S7a) but also that of the CD of one chain (Fig. 4f and Supplementary Fig. S7b,d), or the NBD of one chain (Fig. 4a,e and f and Supplementary Fig. S7c,g), due to the presence of the PBD of the other chain, respectively, as may have been inferred from the close distances between the respective domains. Effects on structural stability due to dimerization are also observed for residue pairs on the NBD of one chain and the CD of the other chain (Fig. 4b,d and f and Supplementary Fig. S7f,h). This finding clearly reflects the long-range aspect to rigidity percolation^32^, as the distance between both domains is at least 50 Å but additional constraints due to dimerization are only placed to model (short-range) non-covalent interactions (i.e., hydrogen bonds, salt bridges, and hydrophobic interactions between the monomers). Notably, however, no change in structural stability due to dimerization is observed for pairs of residues located on either NBD (Fig. 4c,f and Supplementary Fig. 7i).

### Coupling of the opening-closing motions mediated by the CDs

Although we did not find evidence for a *direct* coupling of the opening-closing motion or the structural stability of the two NBDs, the fact that PPDK dissociates in a cold-dependent manner and then becomes inactive^24^ leads to the question as to why only the PPDK dimer is active. Previously, we showed for monomeric PPDK that the CD motion and the opening-closing motion of the NBD is coupled^8^. Above, we showed by CNA that the structural stability of the CD and the NBD of one monomer is influenced by the presence of another monomer (Fig. 4a,b,d,e and f). Taken together, it is tempting to hypothesize that *PPDK* *dimerization influences the opening-closing motions of the NBDs*, and that this influence is *mediated via the CDs of both chains* (Supplementary Fig. S9). Initial support for this hypothesis is found by comparing the PMFs of the opening-closing motion of the NBD, when the CD is near the PBD, in the monomeric state (Δ*G*_open__→__closed_ ~20 kcal mol^−1^; see Fig. 6 in ref. 8) and in the dimeric state (with the CD of the other chain facing the PBD = state a), Δ*G*_open → closed_ ~16 kcal mol^−1^; Fig. 3). The comparison indicates that the closing of the NBD is less energetically unfavorable in the dimeric state, which could explain why the PPDK dimer is the active form. Further support for this hypothesis arises from additional PMF computations of the opening-closing motion of the NBD (chain A) of dimeric PPDK, in which the CD of the same chain faces the PBD but that of chain B faces the NBD (states d) and e)). The PMF of state d) reveals a lower free energy for the closing of the NBD of chain A (by ~6 kcal mol^−1^ compared to states a) - c) at distance_NBD1-NBD3_ ~26 Å; Fig. 3). The ATP-bound conformation of chain B (state e)), which is expected to stabilize the conformation of the NBD and CD of chain B, fosters this effect in that the free energy for the closing of the NBD of chain A is now lower by ~10 kcal mol^−1^ (at distance_NBD1-NBD3_ ~26 Å; Fig. 3) compared to states a) - c). Taken together with our previous results^8^, these findings suggest that the conformational states of the CDs of *both* chains in the PPDK dimer affect the energetics of the opening-closing motions of the two NBDs. However, a thorough validation of this hypothesis would require PMF computations considering both distances_NBD1-NBD3_ as well as two reaction coordinates characterizing the swiveling motion of each CD simultaneously, resulting in a 4D-PMF. Given the computational burden we faced when performing 2D-PMF calculations on monomeric PPDK^8^, such calculations are beyond the scope of the present work, although alternative sampling methods^33^ may alleviate some of this burden.

Finally, our results lead to the question as to why the two NBDs in the asymmetric unit have been found with *distinct* conformational states in the crystal structure PDB ID 5JVJ although both CDs do face the respective PBDs. A qualitative analysis of the crystal packing environments of the NBDs suggests that the crystal packing contributes favorably to, if not fosters, the occurrence of the conformationally distinct states of the NBDs (see section Supplementary Results, Supplementary Table S4, and Supplementary Fig. S8).

### Concluding remarks

Results from unbiased MD simulations, cross-correlation analysis, PCA, PMF computations, and CNA did not provide evidence for a *direct* coupling of the opening-closing motion or the structural stability of the two NBDs in dimeric PPDK. However, we provide results that support the hypothesis that PPDK dimerization does influence the opening-closing motion of the NBDs, and that this influence is mediated via the CDs of both chains. Such an influence would be a prerequisite for an alternate binding change mechanism to occur (Supplementary Fig. S9) and could result in an overall mechanism of dimeric PPDK as displayed in Supplementary Figure S10. To the best of our knowledge, this is the first time that a possible explanation has been suggested as to why only dimeric PPDK is active. The suggestion would add another example to the rare occurrences of asymmetric organization of protein structures, and, as in other cases^19, 34^, the asymmetry would serve a special role, here enzyme activation. Besides further comprehensive PMF computations, it should be interesting to investigate the structural dynamics and function of dimeric PPDK by single molecule spectroscopy, applying, e.g., Förster resonance energy transfer measurements, in order to map the timescales of exchange and the pathways between conformational states^35^.

## Methods

### Generation of all-atom dimeric PPDK structures

The crystal structure of the PPDK dimer with the NBD of chain A in the open and the NBD of chain B in the closed conformation now available from the Protein Data Bank^36^ (PDB ID 5JVJ) was used in all analysis. As 86 residues (9 in chain A and 77 in chain B) out of in total 1748 residues have not been resolved in this structure^8^, a model containing all atoms was generated using the program MODELLER^37^. For this, a sequence alignment was prepared considering the parts resolved in the crystal structure, and the missing parts by the template structure PDB ID 1VBG (NBD open) for chain A and the template structure PDB ID 5JVL (NBD closed) for chain B. Additional models of the PPDK dimer with both NBDs closed, and with both NBDs open, were generated by aligning respective monomers of the 5JVJ structure to the residues of the dimer interface (see Supplementary Table S3).

### Molecular dynamics simulations of dimeric PPDK

MD simulations were performed using established protocols as described previously^8^. In detail, co-crystallized water and ligands were removed from the PPDK dimer structures. Hydrogen atoms were added using REDUCE^38^, flipping side chains of Asn, Gln, and His and assigning ionization states of Asp, Glu, and His when appropriate. These model systems were placed in a truncated octahedral box of TIP3P water^39^ leaving a distance of at least 11 Å between the solute and the border of the box. Counter ions were added to neutralize the systems. All MD simulations were performed with the ff99SB force field^40^ using the Amber suite of programs^41^ and the GPU version of PMEMD^42^. Force field parameters for ATP were obtained from Meagher *et al*.^43^; force field parameters for Mg^2+^ were taken from Åqvist^44^. Bonds containing hydrogen atoms were constrained using the SHAKE algorithm^45^, and long range interactions were treated by the particle mesh Ewald (PME) method^46^. A time step of 2 fs was used. The system was equilibrated by, first, applying harmonic restraints to solute atom positions with force constants of at least 5 kcal mol^−1^ Å^−2^ for 100 steps of steepest descent and 400 steps of conjugate gradient minimization. Second, the temperature of the system was raised from 100 K to 300 K in 50 ps of NVT-MD simulations. Third, 150 ps of NPT-MD simulations were performed to adjust the system density. Finally, the force constants of harmonic restraints were gradually reduced to zero during 250 ps of NVT-MD simulations. Production NVT-MD simulations were carried out at 300 K, using the Berendsen thermostat^47^ and a coupling constant of 0.5 ps. Three independent replicates of MD simulations were performed for each system by spawning production runs after the thermalization at temperatures of 299.9 K, 300.0 K, and 300.1 K respectively. The first 2 ns of each trajectory were omitted from subsequent analyses. All MD simulations are listed in Table 1.

### Principle component analysis

The principle component analysis in Cartesian space was performed on snapshots of MD simulations of the PPDK dimer with an aggregate length of 3.6 µs using CPPTRAJ^48^ in a similar manner as described in ref. 8. In detail, an RMS-fit using the 15% least fluctuating residues was performed prior to the analysis to remove global translational and rotational motion. The coordinate covariance matrix was calculated for all C_α_ atoms. The symmetric matrix was diagonalized by an orthogonal coordinate transformation, yielding the eigenvalues and eigenvectors (principle components). An eigenvalue corresponds to the mean square eigenvector coordinate fluctuation (the variance) and, hence, describes how much a principal component contributes to the total coordinate fluctuations^49^.

To analyze the locality or collectivity of motions for the domains of PPDK, the collectivity index *κ* described in refs 50 and 51 was calculated (equation 3)

3 $$\kappa =\frac{1}{N}\exp \{-\sum _{i=1}^{N}{\Delta }{\vec{r}}_{i}^{2}\,\mathrm{log}\,{\Delta }{\vec{r}}_{i}^{2}\}$$

with *N* being the number of atoms in the (sub)domain, and ${\rm{\Delta }}{\vec{r}}_{i}$ being the relative displacement of the principle component. All values of ${\rm{\Delta }}{\vec{r}}_{i}$ were scaled consistently such that $\sum _{i=1}^{N}{\rm{\Delta }}{\vec{r}}_{i}^{2}=1$. A value of *κ* = 1 indicates a mode of maximal collectivity, that is, all ${\rm{\Delta }}{\vec{r}}_{i}$ are identical. Conversely, if only one atom is affected by the mode, *κ* reaches the minimal value of 1/N.

### Generation of transition paths

For the potential of mean force computations, plausible pathways of the opening-closing motion have been generated in a similar way as described in ref. 8 using targeted normal mode-based geometric simulations by the NMSim approach^25, 26^. In detail, NMSim is a three-step protocol for multiscale modeling of protein conformational changes that incorporates information about preferred directions of protein motions into a geometric simulation algorithm. In the first step, the protein structure is coarse-grained by the software FIRST^52, 53^ into rigid parts connected by flexible links. For this, an energy cut-off for including hydrogen bonds (and salt bridges) of −1 kcal mol^−1^ and a distance cutoff for including hydrophobic constraints of 0.35 Å were used. In the second step, low-frequency normal modes are computed by rigid cluster normal mode analysis (RCNMA) with a 10 Å distance cutoff for considering interactions between C_α_ atoms. In the third step, a linear combination of the first 50 normal modes was used to bias backbone motions along the low-frequency normal modes, while the side chain motions were biased towards favored rotamer states, generating 100 conformations in 100 simulation cycles with a step size of 0.5 Å and side chain distortion of 0.3. Targeted NMSim computations were performed for five states, which vary in the conformation of chain B. For chain A, the open state and the closed state of the NBD was used as start and end conformations for all systems, respectively; the CD of chain A was facing the PBD in all simulations. The start and end conformations of chain B were in state a): NBD in an unbound and open conformation with the CD facing the PBD; in state b): NBD in an unbound and closed conformation with the CD facing the PBD; in state c) NBD in an ATP-bound and closed conformation with the CD facing the PBD; in state d): NBD in an unbound and closed conformation with the CD facing the NBD and in state e): NBD in an ATP-bound and closed conformation with the CD facing the NBD (see Fig. 3a). State c) resembles what has been found in PDB ID 5JVJ.

### Potential of mean force computations

Free energy profiles of the opening-closing motion of the NBD of chain A were computed along the NMSim-generated transition paths with further reference points added by using the conformation after 3 ns of umbrella sampling^54^ as a starting point for the next interval followed by the Weighted Histogram Analysis Method (WHAM)^55^. All umbrella sampling simulations with ATP bound to the NBD of chain B also contained Mg^2+^ in the NBD of chain B. Possible interference between the NBDs was investigated by performing multiple umbrella sampling/PMF calculations of the opening-closing motion of chain A for systems with chain B in states a) - e) (see above). The opening-closing motion of the NBD was analyzed along the reaction coordinate distance_NBD1-NBD3_, measured between S215_Cα_ – E272_Cα_, which has been shown to represent the opening-closing motion of the NBD very well (see Supplementary Fig. 4e in ref. 8). Umbrella sampling MD simulations were performed for distance_NBD1-NBD3_ between 26 Å and 42 Å in intervals of 1 Å, applying harmonic potentials with a force constant of 1 kcal mol^−1^ Å^−2^ to tether the conformations to the respective reference point. This resulted in 17 umbrella sampling simulations, each 20 ns long, excluding the first 1 ns from the WHAM analysis. Approximately Gaussian-shaped frequency distributions were obtained for each reference point along the reaction coordinate, with all such distributions well overlapping (Supplementary Fig. S5). The latter is a prerequisite for the successful application of WHAM to extract a PMF from these distributions. The Monte Carlo bootstrapping analysis implemented in WHAM using 200 resampling trials was applied to establish the errors at the reference points. The umbrella sampling simulations are summarized in Supplementary Table S1.

### Constraint Network Analysis

Constraint Network Analysis (CNA) is a graph theory-based rigidity analysis that links biomolecular structure, (thermo-)stability, and function^27, 29^. In the CNA approach, a protein is represented as a constraint network with bodies (representing atoms) connected by sets of bars (constraints, representing covalent and noncovalent interactions)^52^. A rigid cluster decomposition of the constraint network then identifies rigid parts that are connected by flexible links. By gradually removing noncovalent constraints from an initial network representation of a biomolecule, a succession of network states is generated that yields a ‘constraint dilution trajectory’^30, 56^. Analyzing such a trajectory by rigidity analysis reveals a hierarchy of rigidity that reflects the modular structure of biomolecules in terms of secondary, tertiary, and supertertiary structure.

We applied CNA to investigate the effect of the dimerization on the structural stability of PPDK using three sets of coordinates extracted from one MD simulation of the PPDK dimer: coordinates of only chain A, of only chain B, and of both chains. For this, the first 200 ns of the MD simulation started with the PPDK dimer in the open/closed conformation of the NBDs and equilibrated at 300.0 K was used, where the conformations of the NBDs remain particularly stable (MD simulation started from open/closed and unbound conformation no. 2 shown in Fig. 1b; mean distance_NBD1-NBD3_ of 38.7 Å (SEM ~1.0 Å) and 31.3 Å (SEM ~1.0 Å) for chain A and B, respectively). With CNA, constraint dilution simulations^30, 56^ were performed on ensembles of 10,000 structures each, of which water molecules and counter ions had been stripped off. A stepwise decrease of the energy cutoff *E*_cut_ for including hydrogen bonds from −0.1 kcal mol^−1^ to −6 kcal mol^−1^ in steps of 0.1 kcal mol^−1^ was performed.

### Analysis of CNA results

Stability maps *rc*_*ij*_ were introduced in ref. 56 to characterize the local rigidity of a biomolecule. A stability map depicts rigid contacts (*rc*) for each residue pair (*i* and *j*), represented by the C_α_ atom, respectively. A rigid contact exists if the two residues belong to the same rigid cluster^57^. Notably, this stability information is not only a qualitative by also quantitative measurement. By performing constraint dilution simulations^31^, each rigid contact is associated with an energy *E*_cut_ at which this rigid contact is lost.

### Data availability

All data generated or analysed during this study are included in this published article (and its Supplementary Information files).

## Electronic supplementary material

Supplementary Information

## References

[1] Andrews TJ, Hatch MD (1969). Properties and mechanism of action of pyruvate, phosphate dikinase from leaves. Biochemical Journal.

[2] Varela-Gómez M, Moreno-Sánchez R, Pardo JP, Perez-Montfort R (2004). Kinetic mechanism and metabolic role of pyruvate phosphate dikinase from Entamoeba histolytica. Journal of Biological Chemistry.

[3] Spronk AM, Yoshida H, Wood HG (1976). Isolation of 3-phosphohistidine from phosphorylated pyruvate, phosphate dikinase. Proceedings of the National Academy of Sciences U. S. A..

[4] Milner Y, Wood HG (1972). Isolation of a pyrophosphoryl form of pyruvate, phosphate dikinase from Propionibacteria. Proceedings of the National Academy of Sciences U. S. A..

[5] Goss NH, Evans CT, Wood HG (1980). Pyruvate phosphate dikinase: sequence of the histidyl peptide, the pyrophosphoryl and phosphoryl carrier. Biochemistry.

[6] Carroll LJ, Xu Y, Thrall SH, Martin BM, Dunaway-Mariano D (1994). Substrate binding domains in pyruvate phosphate dikinase. Biochemistry.

[7] Herzberg O (1996). Swiveling-domain mechanism for enzymatic phosphotransfer between remote reaction sites. Proceedings of the National Academy of Sciences.

[8] Minges ARM (2017). Structural intermediate and directionality of the swiveling motion of PPDK. Scientific Reports.

[9] McGuire M (1996). Determination of the nucleotide binding site within Clostridium symbiosum pyruvate phosphate dikinase by photoaffinity labeling, site-directed mutagenesis, and structural analysis. Biochemistry.

[10] Wei M, Li Z, Ye D, Herzberg O, Dunaway-Mariano D (2000). Identification of domain-domain docking sites within Clostridium symbiosum pyruvate phosphate dikinase by amino acid replacement. J. Biol. Chem..

[11] Ye D (2001). Investigation of the catalytic site within the ATP-grasp domain of Clostridium symbiosum pyruvate phosphate dikinase. Journal of Biological Chemistry.

[12] Herzberg O (2002). Pyruvate site of pyruvate phosphate dikinase: crystal structure of the enzyme-phosphonopyruvate complex, and mutant analysis. Biochemistry.

[13] Cosenza LW, Bringaud F, Baltz T, Vellieux FMD (2002). The 3.0 A resolution crystal structure of glycosomal pyruvate phosphate dikinase from Trypanosoma brucei. Journal of Molecular Biology.

[14] Nakanishi T, Nakatsu T, Matsuoka M, Sakata K, Kato H (2005). Crystal structures of pyruvate phosphate dikinase from maize revealed an alternative conformation in the swiveling-domain motion. Biochemistry.

[15] Fawaz MV, Topper ME, Firestine SM (2011). The ATP-grasp enzymes. Bioorg. Chem..

[16] Hatch M (1979). Regulation of C4 photosynthesis: factors affecting cold-mediated inactivation and reactivation of pyruvate, Pi dikinase. Functional Plant Biology.

[17] Sugiyama T (1973). Purification, molecular, and catalytic properties of pyruvate phosphate dikinase from the maize leaf. Biochemistry.

[18] Shirahashi K, Hayakawa S, Sugiyama T (1978). Cold lability of pyruvate, orthophosphate dikinase in the maize leaf. Plant Physiology.

[19] Goodsell DS, Olson AJ (2000). Structural symmetry and protein function. Annual Review of Biophysics.

[20] Boyer PD (1993). The binding change mechanism for ATP synthase—some probabilities and possibilities. Biochimica et Biophysica Acta - Bioenergetics.

[21] Abrahams JP, Leslie AG, Lutter R, Walker JE (1994). Structure at 2.8 A resolution of F1-ATPase from bovine heart mitochondria. Nature.

[22] Korolev S, Hsieh J, Gauss GH, Lohman TM, Waksman G (1997). Major domain swiveling revealed by the crystal structures of complexes of E. coli Rep helicase bound to single-stranded DNA and ADP. Cell.

[23] Krissinel E, Henrick K (2007). Protein interfaces, surfaces and assemblies service PISA at European Bioinformatics Institute. Journal of Molecular Biology.

[24] Usami S, Ohta S, Komari T, Burnell JN (1995). Cold stability of pyruvate, orthophosphate dikinase of Flaveria brownii. Plant Molecular Biology.

[25] Ahmed A, Rippmann F, Barnickel G, Gohlke H (2011). A normal mode-based geometric simulation approach for exploring biologically relevant conformational transitions in proteins. Journal of Chemical Information and Modeling.

[26] Krüger DM, Ahmed A, Gohlke H (2012). NMSim web server: integrated approach for normal mode-based geometric simulations of biologically relevant conformational transitions in proteins. Nucleic Acids Research.

[27] Pfleger C, Rathi PC, Klein DL, Radestock S, Gohlke H (2013). Constraint Network Analysis (CNA): a Python software package for efficiently linking biomacromolecular structure, flexibility, (thermo-)stability, and function. Journal of Chemical Information and Modeling.

[28] Jacobs & Thorpe. (1995). Generic rigidity percolation: The pebble game. Physical Review Letters.

[29] Hermans, S., Pfleger, C., Nutschel, C., Hanke, C. & Gohlke, H. Rigidity theory for biomolecules: Concepts, software, and applications. *WIREs Computational Molecular Science* **7**, e1311 (2017).

[30] Radestock S, Gohlke H (2008). Constraint Network Analysis: Exploiting the Link Between Protein Rigidity and Thermostability. Engineering in Life Sciences.

[31] Pfleger C, Radestock S, Schmidt E, Gohlke H (2013). Global and local indices for characterizing biomolecular flexibility and rigidity. Journal of Computational Chemistry.

[32] Thorpe M, Jacobs D, Djordjevic B (1996). Generic Rigidity Percolation. Condensed Matter Theories.

[33] Piana S, Laio A (2007). A bias-exchange approach to protein folding. The journal of physical chemistry B.

[34] Swapna LS, Srikeerthana K, Srinivasan N (2012). Extent of structural asymmetry in homodimeric proteins: prevalence and relevance. PloS one.

[35] Dimura M (2016). Quantitative FRET studies and integrative modeling unravel the structure and dynamics of biomolecular systems. Current Opinion in Structural Biology.

[36] Bernstein FC (1977). The Protein Data Bank. A computer-based archival file for macromolecular structures. European Journal of Biochemistry.

[37] Eswar, N. *et al*. Comparative protein structure modeling using MODELLER. *Current Protocols in Protein Science* Chapter 2, Unit 2.9 (2007). 10.1002/0471140864.ps0209s50 18429317

[38] Word JM, Lovell SC, Richardson JS, Richardson DC (1999). Asparagine and glutamine: using hydrogen atom contacts in the choice of side-chain amide orientation. Journal of Molecular Biology.

[39] Jorgensen WL, Chandrasekhar J, Madura JD, Impey RW, Klein ML (1983). Comparison of simple potential functions for simulating liquid water. Journal of Chemical Physics.

[40] Hornak V (2006). Comparison of multiple Amber force fields and development of improved protein backbone parameters. Proteins.

[41] Case DA (2005). The Amber biomolecular simulation programs. Journal of Computational Chemistry.

[42] Salomon-Ferrer R, Götz AW, Poole D, Le Grand S, Walker RC (2013). Routine microsecond molecular dynamics simulations with AMBER on GPUs. 2. Explicit solvent particle mesh Ewald. Journal of Chemical Theory and Computation.

[43] Meagher KL, Redman LT, Carlson HA (2003). Development of polyphosphate parameters for use with the AMBER force field. Journal of Computational Chemistry.

[44] Aqvist J (1990). Ion-water interaction potentials derived from free energy perturbation simulations. Journal of Physical Chemistry.

[45] Ryckaert J-P, Ciccotti G, Berendsen HJ (1977). Numerical integration of the cartesian equations of motion of a system with constraints: molecular dynamics of n-alkanes. Journal of Chemical Physics.

[46] Cheatham TE, Miller J, Fox T, Darden T, Kollman P (1995). Molecular dynamics simulations on solvated biomolecular systems: the particle mesh Ewald method leads to stable trajectories of DNA, RNA, and proteins. Journal of the American Chemical Society.

[47] Berendsen HJ, van Postma J, van Gunsteren WF, DiNola A, Haak J (1984). Molecular dynamics with coupling to an external bath. Journal of Chemical Physics.

[48] Roe DR, Cheatham TE (2013). PTRAJ and CPPTRAJ: software for processing and analysis of molecular dynamics trajectory data. Journal of Chemical Theory and Computation.

[49] Hayward, S. & De Groot, B. L. Normal modes and essential dynamics. *Molecular Modeling of Proteins*, 89–106 (2008). 10.1007/978-1-59745-177-2_5 18446283

[50] Ahmed A, Villinger S, Gohlke H (2010). Large‐scale comparison of protein essential dynamics from molecular dynamics simulations and coarse‐grained normal mode analyses. Proteins: Structure, Function, and Bioinformatics.

[51] Brüschweiler R (1995). Collective protein dynamics and nuclear spin relaxation. Journal of Chemical Physics.

[52] Jacobs DJ, Rader AJ, Kuhn LA, Thorpe MF (2001). Protein flexibility predictions using graph theory. Proteins.

[53] Thorpe MF, Lei M, Rader AJ, Jacobs DJ, Kuhn LA (2001). Protein flexibility and dynamics using constraint theory. Journal of Molecular Graphics and Modelling.

[54] Torrie GM, Valleau JP (1977). Nonphysical sampling distributions in Monte Carlo free-energy estimation: Umbrella sampling. Journal of Computational Physics.

[55] Kumar S, Rosenberg JM, Bouzida D, Swendsen RH, Kollman PA (1992). The weighted histogram analysis method for free-energy calculations on biomolecules. I. The method. Journal of Computational Chemistry.

[56] Radestock S, Gohlke H (2011). Protein rigidity and thermophilic adaptation. Proteins.

[57] Rathi, P. C., Jaeger, K.-E. & Gohlke, H. Structural Rigidity and Protein Thermostability in Variants of Lipase A from *Bacillus subtilis*. *PLoS ONE* **10**, e0130289 (2015). 10.1371/journal.pone.0130289 PMC4493141 26147762

