# Supplementary material for: On the potential alternate binding change mechanism in a dimeric structure of Pyruvate Phosphate Dikinase
Source: Sci Rep. 2017 Aug 14;7:8020. doi: 10.1038/s41598-017-08521-w (PMC5556012; doi:10.1038/s41598-017-08521-w)
Supplement: Supplementary file 1 — Supplementary Information [file 41598_2017_8521_MOESM1_ESM.pdf]

Supplementary Information

On the potential alternate binding change mechanism in a dimeric structure of Pyruvate Phosphate Dikinase

Daniel Ciupka[1], Holger Gohlke[1,2*]

[1]Institute of Pharmaceutical and Medicinal Chemistry, Heinrich-Heine-Universität Düsseldorf, 40225 Düsseldorf, Germany.

[2]John von Neumann Institute for Computing (NIC), Jülich Supercomputing Centre (JSC) & Institute for Complex Systems - Structural Biochemistry (ICS 6), Forschungszentrum Jülich, 52425 Jülich, Germany.

Corresponding Author

*Phone: +49(0)221-81-13662. *Fax: +49(0)221-81-13847. E-mail: gohlke@uni-duesseldorf.de; h.gohlke@fz-juelich.de

TABLE OF CONTENTS

SUPPLEMENTARY RESULTS 3
CRYSTAL PACKING ENVIRONMENTS OF THE NBDS IN PDB ID 5JVJ 3
SUPPLEMENTARY FIGURES 4
FIGURE S1: CLUSTERS OF PPDK CRYSTAL STRUCTURES 4
FIGURE S2: VALIDATION OF THE DIMER INTERFACE 5
FIGURE S3: PCA DETAILS 6
FIGURE S4: CROSS-CORRELATIONS BETWEEN THE PBDS 7
FIGURE S5: OVERLAP OF UMBRELLA SAMPLING SIMULATIONS 8
FIGURE S7: EFFECT OF THE DIMERIZATION ON THE STABILITY OF PPDK 10
FIGURE S8: CRYSTAL CONTACTS INVOLVING THE NBDS 11
FIGURE S9: SUGGESTED COUPLING OF MOTIONS AND STRUCTURAL STABILITY IN THE PPDK DIMER 12
FIGURE S10: PROPOSED MECHANISM OF DIMERIC PPDK 13
SUPPLEMENTARY TABLES 14
TABLE S1: UMBRELLA SAMPLING MD SIMULATIONS FOR PMF COMPUTATIONS. 14
TABLE S2: ANALYSIS OF THE PPDK INTERFACES BY PDBEPISA 15
TABLE S3: CONSERVATION OF DIMER INTERFACE RESIDUES. 16
TABLE S4: CRYSTAL CONTACTS INVOLVING THE NBD IN PDB ID 5JVJ 18
SUPPLEMENTARY REFERENCES 19

Supplementary Results

Crystal packing environments of the NBDs in PDB ID 5JVJ

The PDBePISA tool [1] reveals six symmetry-related interfaces involving the NBD (Supplementary Table S2, interfaces labeled in italics), forming two different crystal packing environments for the NBDs (Supplementary Table S4). We next probed if the observed crystal packing would also be possible with the respective alternative conformational state of the NBD by superimposing an all-residue model of the open conformation of the NBD onto the closed conformation, and *vice versa*, using the coordinates of the third NBD subdomain (aa 244–340) for overlaying. Excluding crystal contacts where the positions of the interacting atoms in the alternative NBD conformations differ by > 1 Å, or where positions of interacting atoms can be maintained by sidechain rotations or motions of flexible loops, the analysis reveals that 12 crystal contacts for chain A (open conformation) and 2 crystal contacts for chain B (closed conformation) would break if the respective alternative conformation of the NBD were located there, rather than the one found in the crystal structure (Supplementary Fig. S8 and Supplementary Table S4, labeled in bold). While this analysis is qualitative only, it suggests that the crystal packing contributes favorably to, if not fosters, the occurrence of conformationally different states of the NBDs in the asymmetric unit. Notable support for the influence of crystal packing on the conformational state of that PPDK structure arises from the fact that for chain B with a closed and likely adenine nucleotide-bound NBD, the CD is located close to the PBD, rather than the NBD (Fig 1b) [2]. This arrangement is unexpected in view of the enzyme mechanism [2] and leads to an energetically unfavorable conformational state of that chain (see Fig. 6 in ref. [2]).

Supplementary Figures

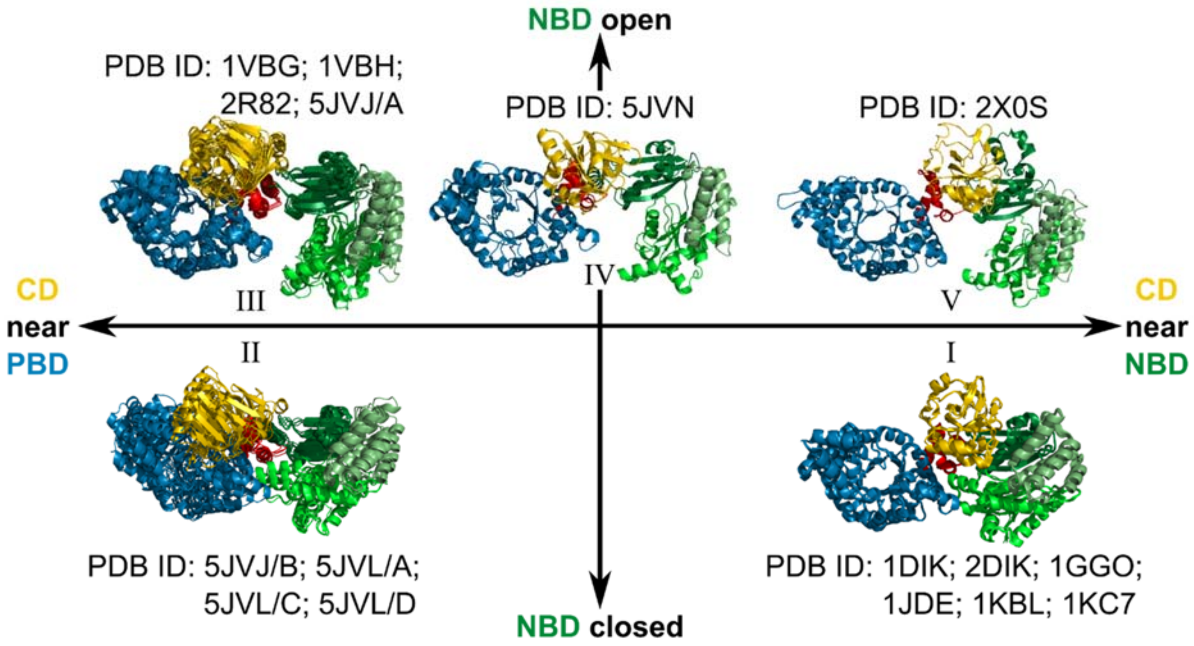

Figure S1: Clusters of PPDK crystal structures adapted from ref. [2], showing the CD near the PBD (left), between PBD and NBD (middle), or near the NBD (right), and the NBD in an open (top) or closed (bottom) conformation.

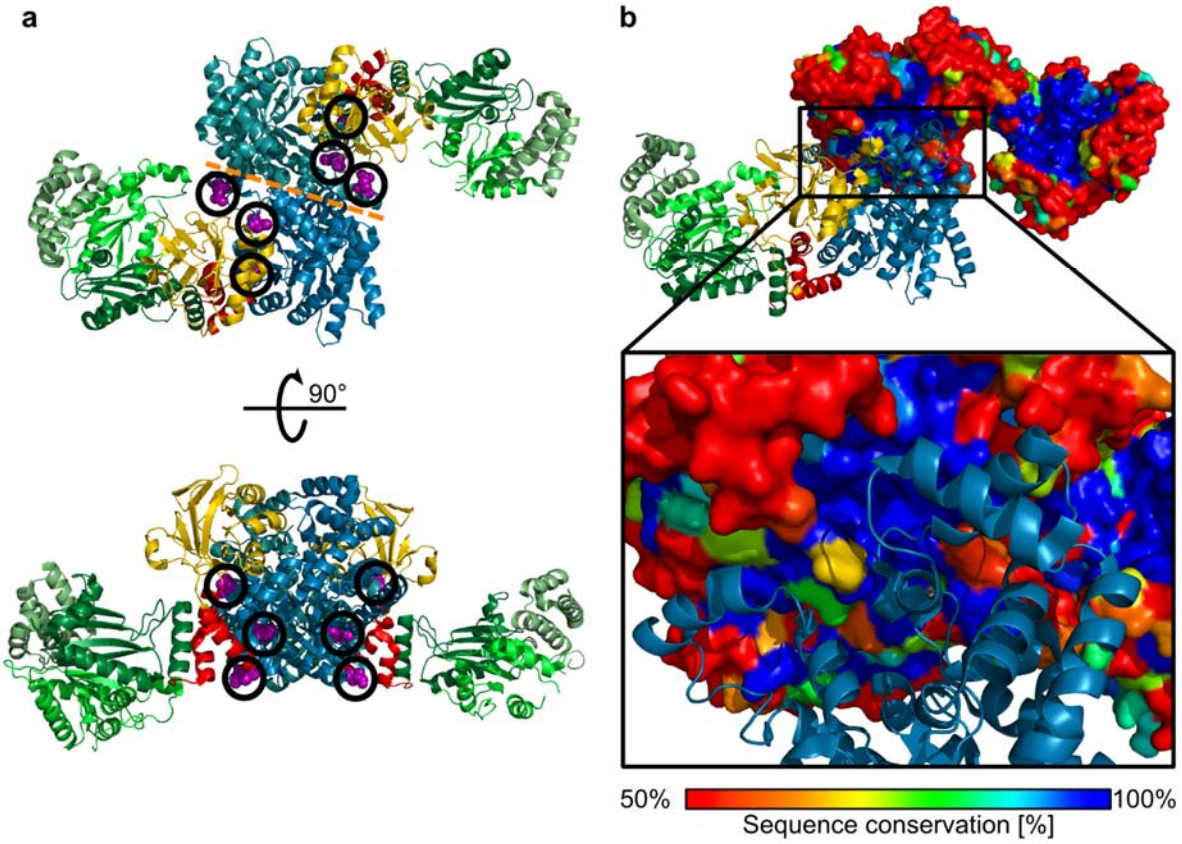

Figure S2: Validation of the dimer interface. (a) Crystal structure PDB ID 5JVJ showing mutations (spheres in purple and circled) reported to reduce PPDK's cold-dependent dissociation and thereby inactivation [3] with the dimer interface indicated by the dashed orange line in top view (top) and side view (bottom). **(b)** All atom model of PDB ID 5JVJ showing the degree of sequence conservation (see color scale) mapped onto the surface of chain B of the whole structure (top) and as a close-up of the dimer interface. The conservation degree was computed from a multiple sequence alignment obtained with MAFFT [4] of 1000 PPDK sequences identified by BLASTp [5] in the NCBI-NR database of non-redundant protein sequences [6].

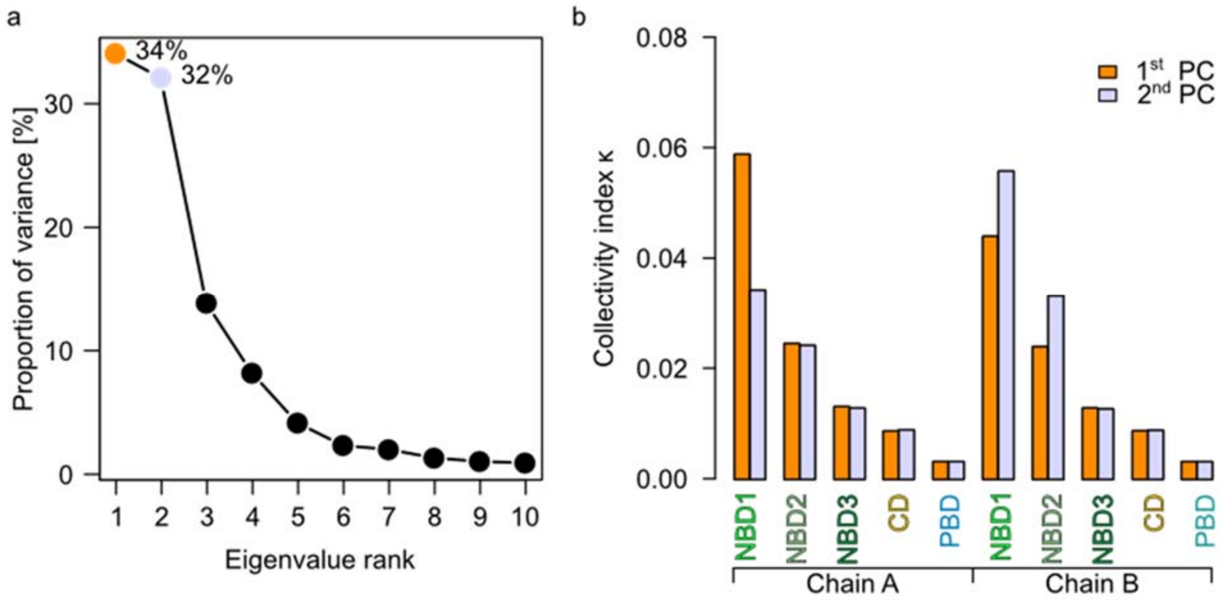

Figure S3: PCA details of the PPDK dimer. (a) The proportion of explained variance is depicted *versus* the number of the principal component (PC) ranked by its eigenvalue. The 1st (gold) and 2nd (silver) PC relate to Figure 2a. 66% of the variance can be explained by the first two PCs. **(b)** (Sub)Domain-wise collectivity index κ of the 1st and 2nd PC.

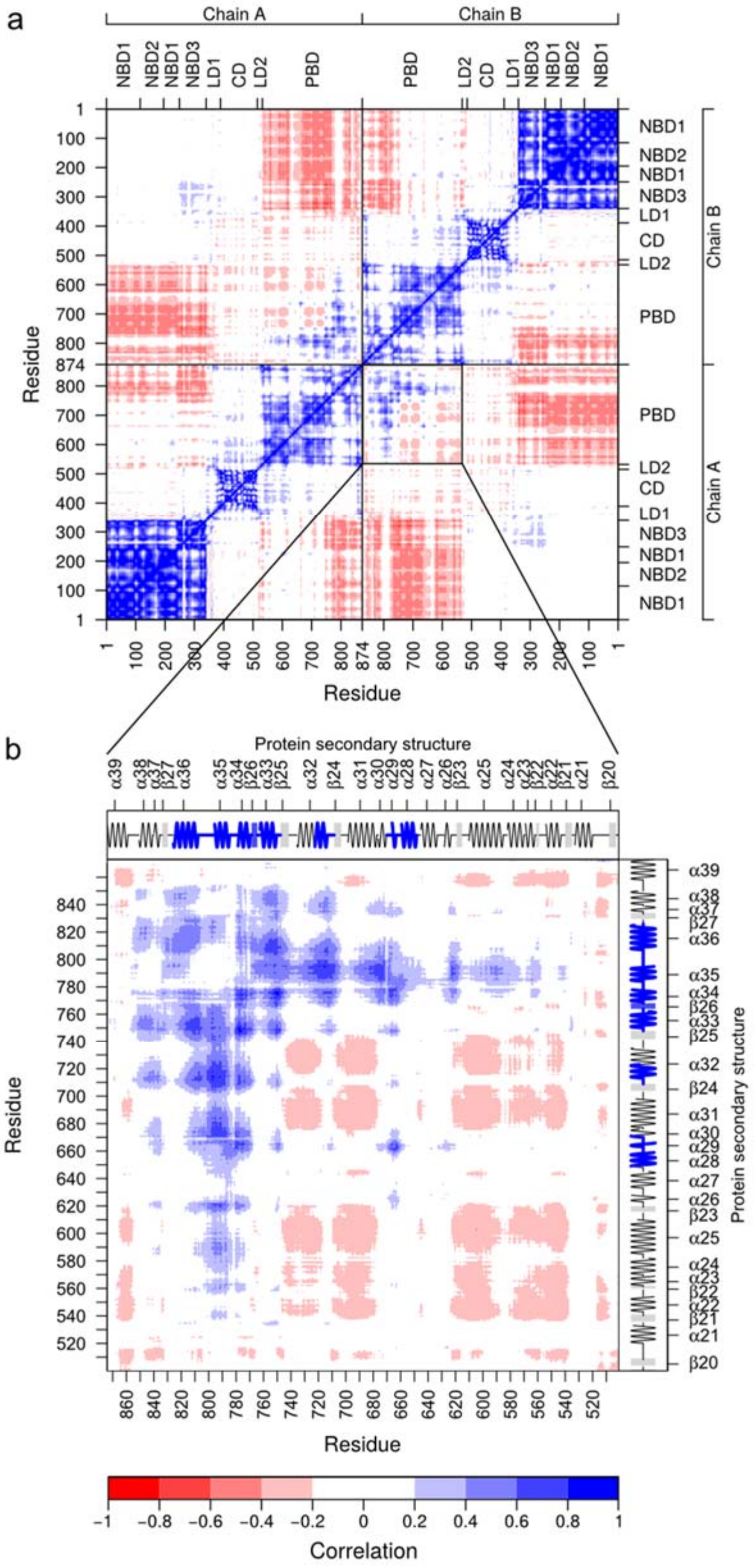

Figure S4: Cross-correlations between the PBDs. (a) Cross-correlation map of C_α atom fluctuations of the PPDK dimer from PDB ID 5JVJ (the plot is identical to Fig. 2c). **(b)** Blow-up of the region displaying intermolecular correlations between the PBDs of chains A and B, with the secondary structures as predicted by DSSP [7] in black/gray and the interface residues in blue labeled on the top and right axes.

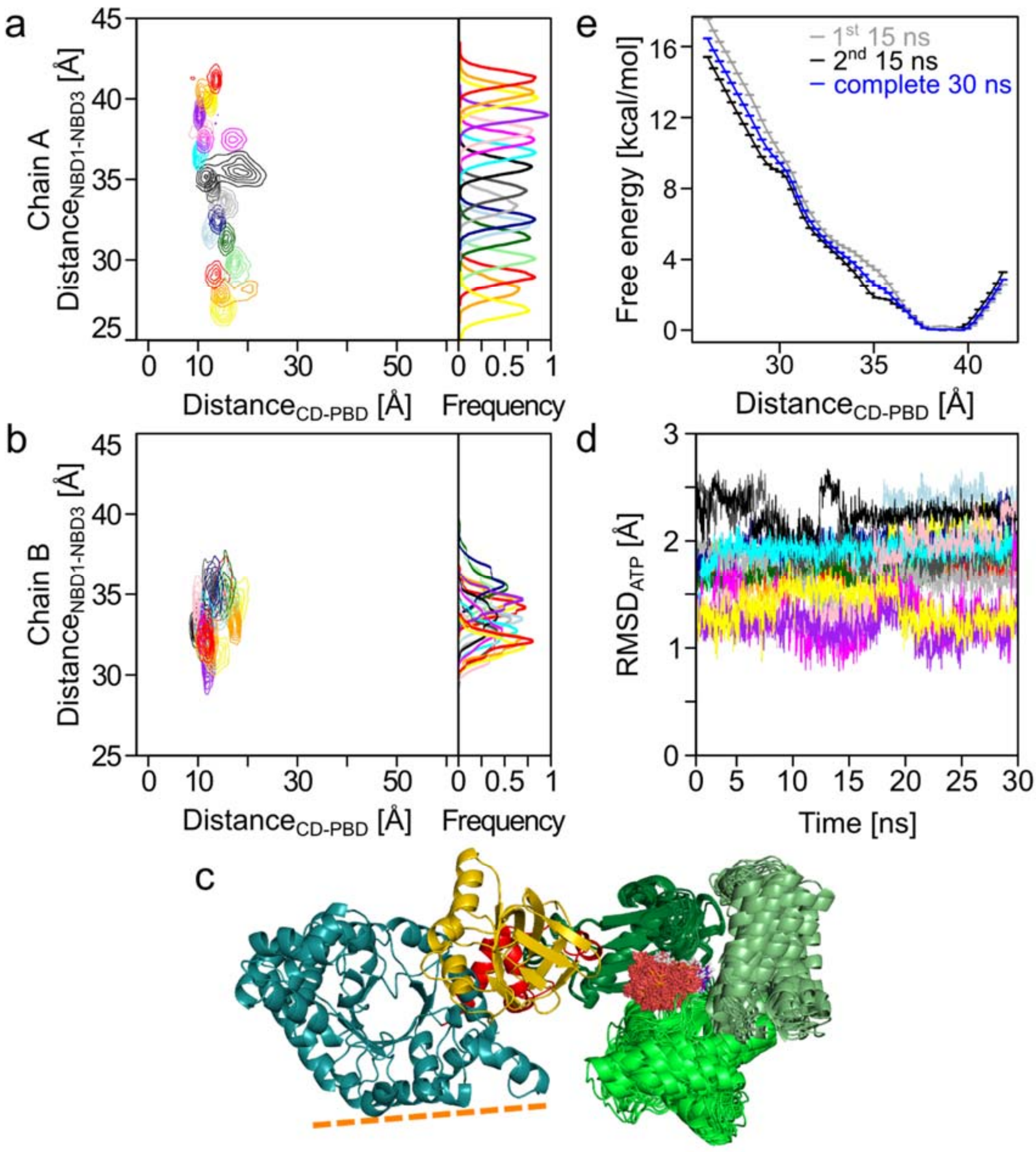

Figure S5: Overlap of umbrella sampling simulations, exemplarily shown for state c) (Fig. 3 in the main text) and chain A **(a)** and B **(b)** in terms of frequency distributions of the distance$_{NBD1\text{-}NBD3}$ between S215$_{C\alpha}$ – E272$_{C\alpha}$, (used as a reaction coordinate in the case of chain A), and distance$_{CD\text{-}PBD}$ between H565$_{C\alpha}$ – H456$_{C\alpha}$, which was not restrained during the simulations. The umbrella sampling simulations display well-overlapping frequency distributions of distance$_{NBD1\text{-}NBD3}$ for chain A. **(c)** Overlay of the NBD and ATP coordinates to an all-atom model of PDB ID 5JVJ. For clarity, only chain B is displayed; the dimer interface is indicated by the orange line; the state of the opening-closing motion is shown for each umbrella sampling window by an overlay of the closest-to-the-average structures of the NBD. The ATP coordinates are shown for every 20 ps for all umbrella sampling simulations of state c. **(d)** RMSD of ATP within the binding site with respect to the starting structure. **(e)** Convergence of the PMF, computed for the first 15 ns (gray), the second 15 ns (black), and the complete 30 ns (blue) simulation time per window.

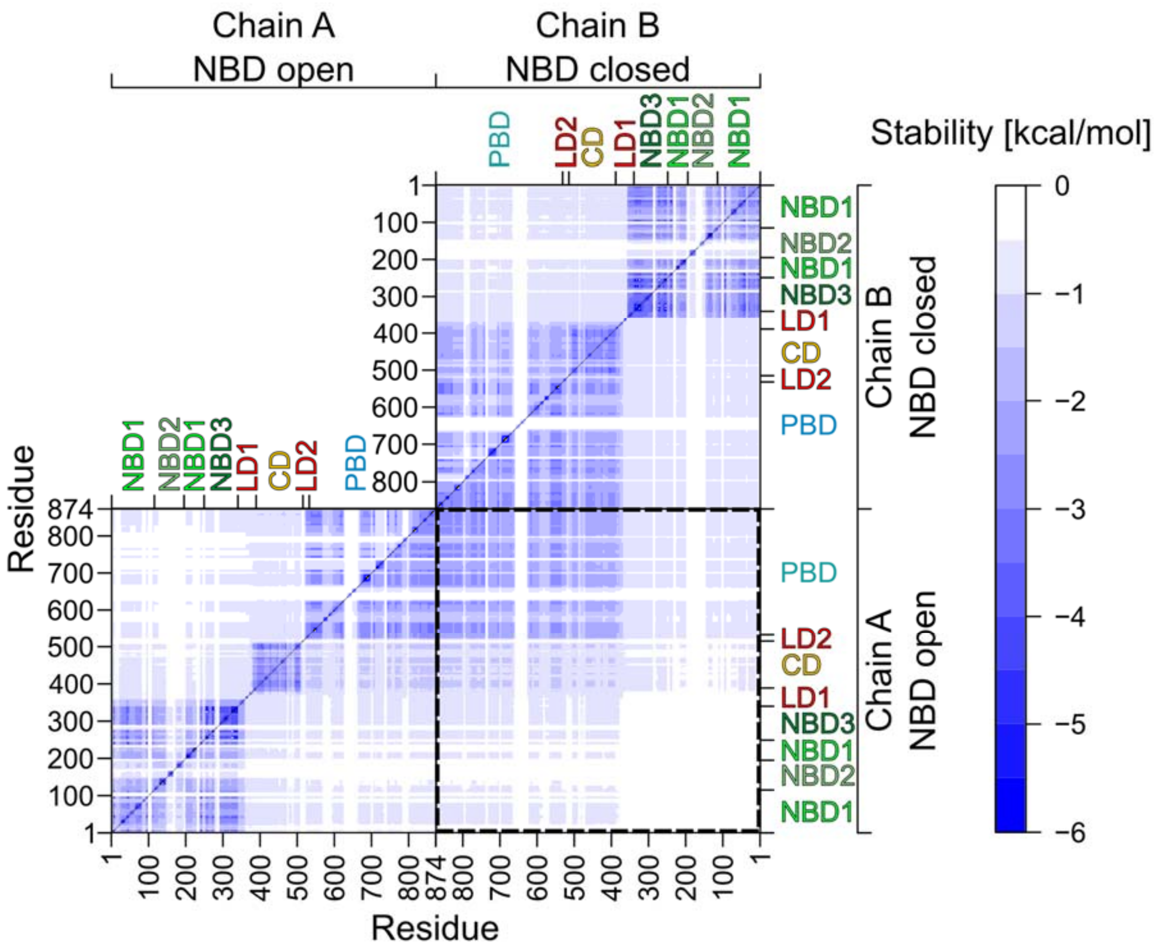

Figure S6: Structural stability of monomeric and dimeric PPDK, computed with CNA. Stability maps depicting the energy E_{cut} (see color scale at the right) at which a rigid contact is lost between two residues along a constraint dilution trajectory of the PPDK dimer (bottom triangle), the monomer with open NBD (chain A, bottom left triangle), and the monomer with closed NBD (chain B, top left triangle). The residue numbering is given on the left and on the bottom, and the protein domains are labeled on the top and on the right.

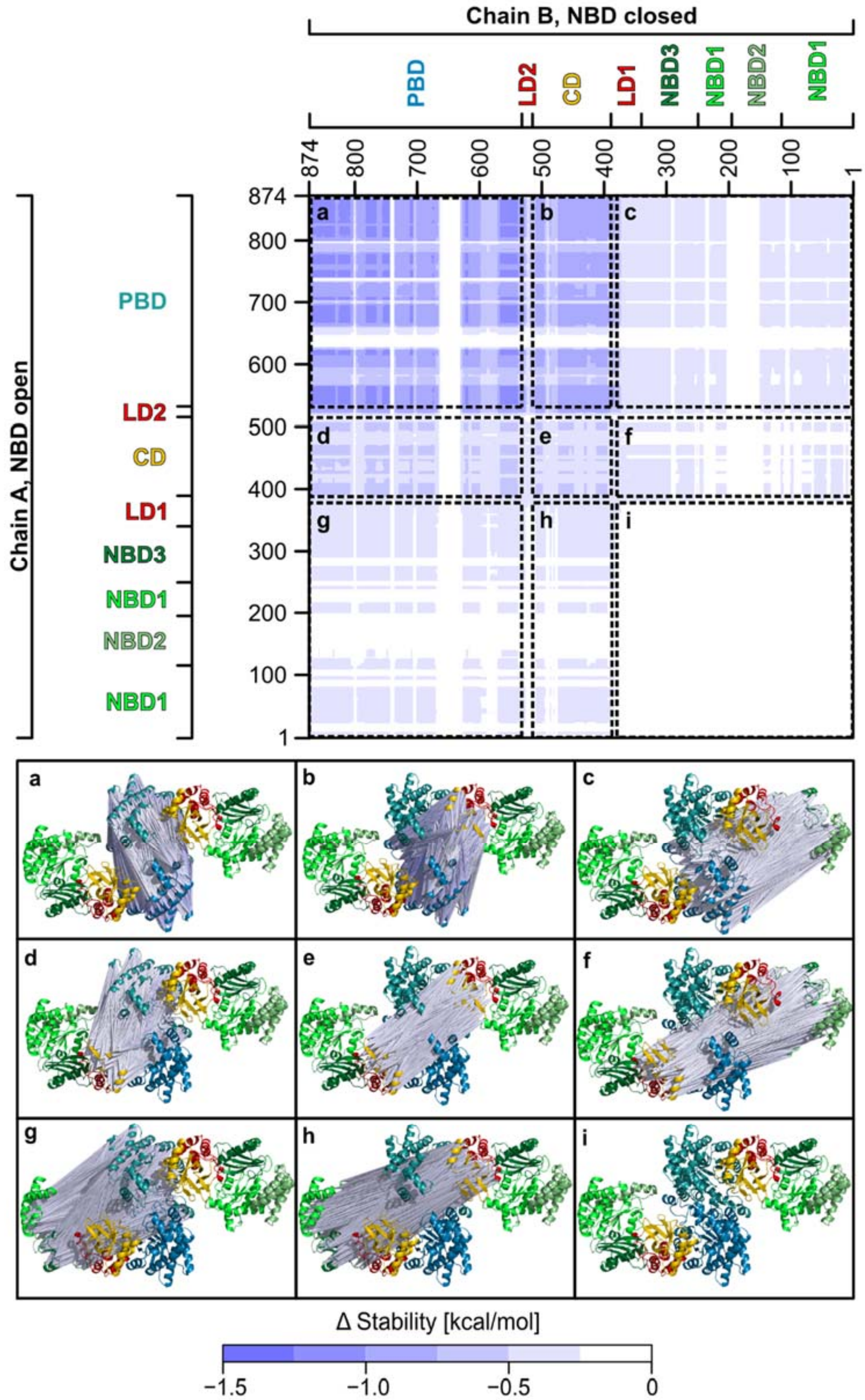

Figure S7: Effect of the dimerization on the stability of PPDK as computed by CNA. Difference in the stability of rigid contacts in terms of (top) a stability map with the residue numbering and labels for the domains given on the left and on the bottom, and (bottom) as lines connecting the C_α atoms of two residues of the PPDK structure 5JVJ (with missing atoms added by homology modeling): Between **(a)** PBD and PBD, **(b)** PBD and CD, **(c)** PBD and NBD, **(d)** CD and PBD, **(e)**, CD and CD, **(f)** CD and NBD, **(g)** NBD and PBD, **(h)** NBD and CD, and **(i)** NBD and NBD of chain A and chain B, respectively. Rigid contacts that are more stable in the dimer than in the monomer are indicated in darker blue colors, see color scale at the bottom.

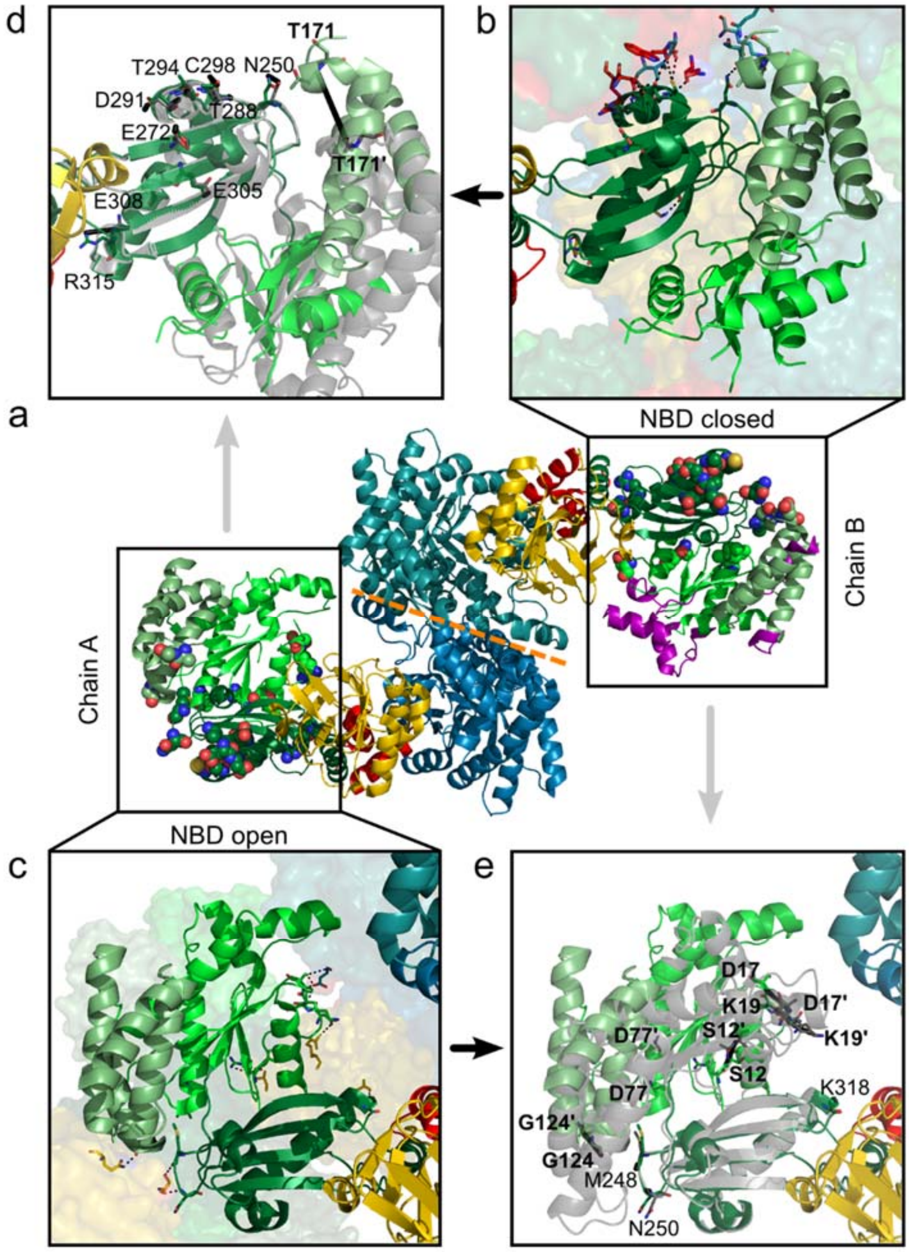

Figure S8: Crystal contacts involving the NBDs of the PPDK structure PDB ID 5JVJ. **(a)** Cartoon representation of the PPDK dimer (including all atoms) with the PBD colored blue, the CD colored yellow, the NBD depicted by three different greens, the dimer interface indicated by the dashed orange line, and residues forming crystal contacts shown as spheres. Residues not resolved in the structure were modeled and are displayed in purple. **(b, c)** Detailed view on chain B (closed NBD, **(b)**) and chain A (open NBD, **(c)**) with residues forming crystal contacts shown as sticks. **(d, e)** Superposition of the open and closed conformation of the NBD of chain A **(d)** and B **(e)**, respectively. The respective alternative NBD conformation is shown in gray, with the distance of respective atoms involved in forming crystal contacts shown as black lines.

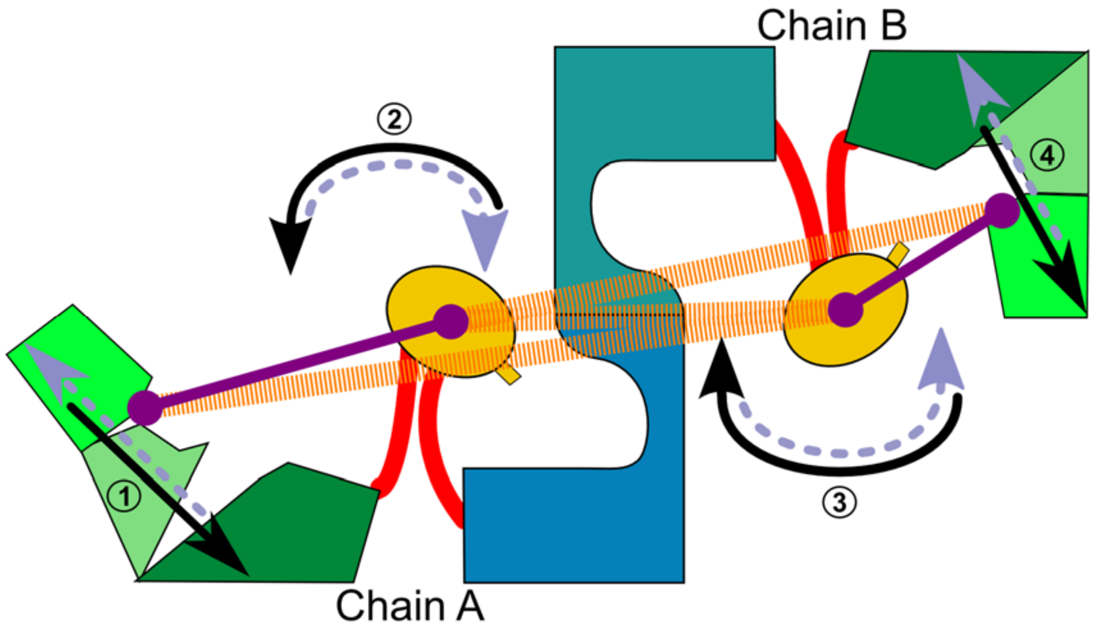

Figure S9: Suggested coupling of motions and structural stability in the PPDK dimer. Intramolecular coupling of the swiveling motion of the CD with the opening-closing motion of the NBD (indicated by deep purple bars) was demonstrated for the PPDK monomer [2]. The results from the present study suggest furthermore that PPDK dimerization influences the opening-closing motion of an NBD *mediated via the CDs of both chains*. Mutual increases in structural stability due to dimerization as revealed by CNA are indicated by orange dashed lines. Our findings lend support to the hypothesis of an alternate binding change mechanism in the PPDK dimer: When the NBD of chain A is closing **(1)**, the CD of chain A swivels towards the NBD **(2)**, the CD of chain B swivels towards the PBD **(3)**, and the NBD of chain B is opening **(4)** (illustrated by black arrows) and *vice versa* (illustrated by gray arrows).

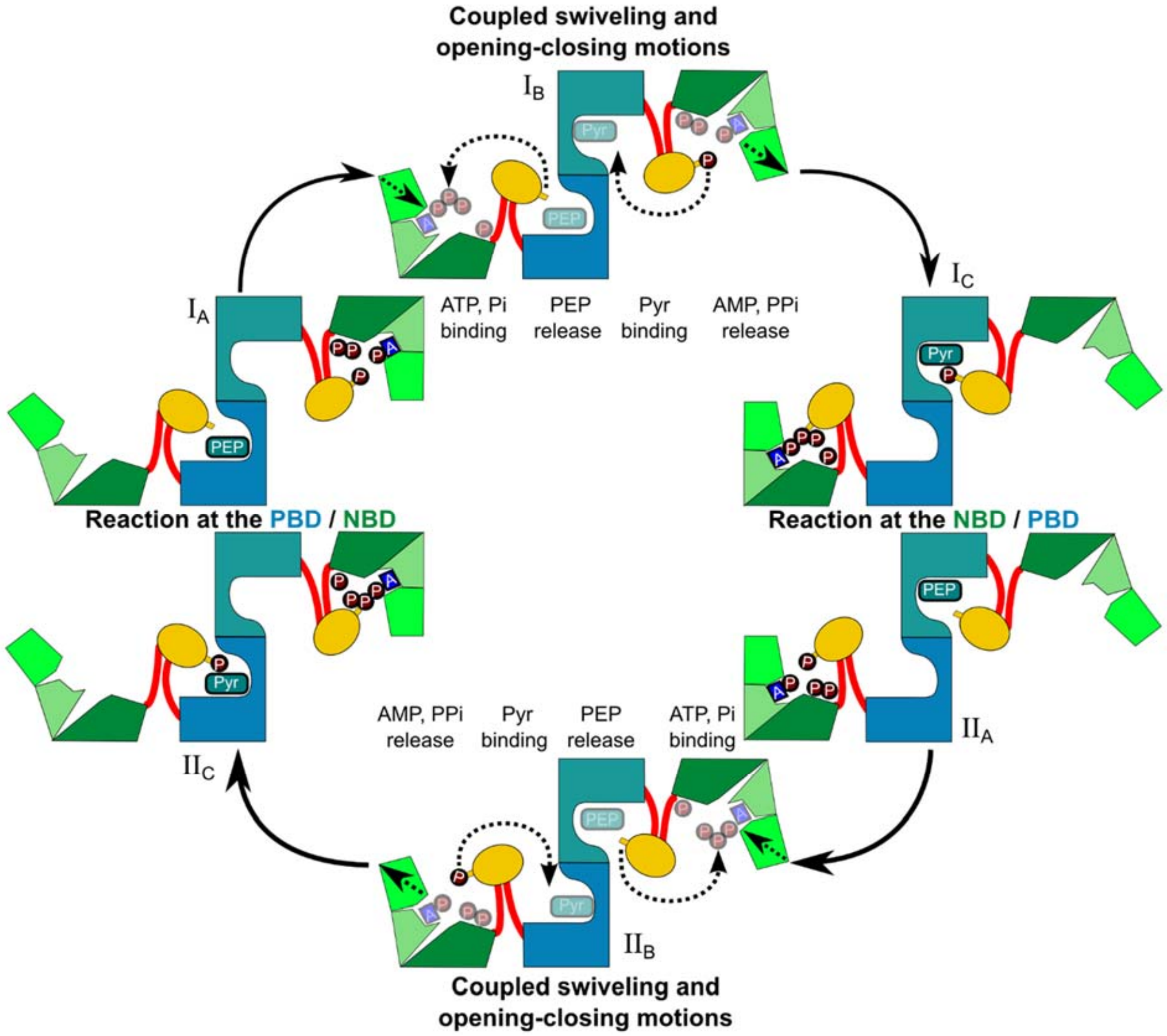

Figure S10: Proposed mechanism of dimeric PPDK. The proposed mechanism involves the following steps: ($II_C \rightarrow I_A$) Transfer of a phosphoryl group from ATP to H456 of the CD of chain B. (I_B) ATP binding to the NBD of chain A leads to the closing of this NBD, which is coupled with the CD motion of chain A swiveling from the PBD to the NBD, and the CD motion of chain B swiveling from the NBD to the PBD; the latter CD transports the phosphoryl group from the NBD to the PBD of chain B. ($I_C \rightarrow II_A$) The phosphoryl group bound to H456 of the CD of chain B is transferred to pyruvate at the PBD of chain B to generate PEP. Steps $II_{A\text{-}C}$ are identical to $I_{A\text{-}C}$ but now the functional roles of chains A and B are exchanged. Between II_C and I_A as well as I_C and II_A, reactions (1) and (2) mentioned in the main text take place.

Supplementary Tables

Table S1: Umbrella sampling MD simulations for PMF computations.

State	Conformation chain B	Windows	Simulation length [a]
a)	CD facing PBD, NBD open, unbound	17	30 (510)
b)	CD facing PBD, NBD closed, unbound	17	30 (510)
c)	CD facing PBD, NBD closed, ATP bound	17	30 (510)
d)	CD facing NBD, NBD closed, unbound	17	30 (510)
e)	CD facing NBD, NBD closed, ATP bound	17	30 (510)

[a] For each window (total simulation time for all windows), in ns.

Table S2: Analysis of the PPDK interfaces by PDBePISA[a].

Structure 1			Structure 2		Interface					
Range	$^iN_{res}$ [b]	Symmetry operation	Range	$^iN_{res}$ [b]	Interface area [c]	Δ^iG [d]	Δ^iG P-value	N_{HB} [e]	N_{SB} [f]	CSS [g]
**B PBD**	**56**	**x, y, z**	**A PBD**	**53**	**2077.4**	**□ 17.4**	**0.370**	**34**	**10**	**0.541**
B LD NBD3	*36*	*-x-1/2, y-1/2, -z*	*B LD NBD3*	*42*	*1180*	*□ 10.6*	*0.344*	*13*	*3*	*0*
A PBD CD	44	-x, y, -z+1	A PBD CD	43	1145.8	□6.6	0.443	10	0	0
A CD	*19*	*-x+1/2, y-1/2, -z+1*	*A NBD2-3*	*23*	*440.2*	*□2.4*	*0.500*	*4*	*2*	*0*
A CD	*12*	*x, y-1, z*	*A NBD1, 3*	*14*	*364.4*	*□0.9*	*0.578*	*3*	*5*	*0*
B PBD	*8*	*x, y-1, z*	*B NBD1*	*8*	*203.2*	*□2.2*	*0.403*	*4*	*0*	*0*
B PBD	4	-x, y, -z	B PBD	4	161.3	□ 1.8	0.590	2	0	0
A PBD	*3*	*x-1/2, y-1/2, z*	*A NBD2*	*5*	*102.3*	*□2.1*	*0.615*	*0*	*0*	*0*
B NBD2	*4*	*x-1/2, y-1/2, z*	*B PBD*	*3*	*94.8*	*□1.1*	*0.501*	*0*	*0*	*0*

[a] The dimer interface of PPDK is shown in bold; interfaces involving the NBD are shown in italics.
[b] Number of interface residues.
[c] In $Å^2$.
[d] Solvation free energy gain upon formation of the interface, in kcal mol^{-1}.
[e] Number of hydrogen bonds.
[f] Number of salt bridges.
[g] Complexation significance score.

Table S3: Conservation of dimer interface residues.

<table>
<thead>
<tr><th>Interface residues[a]</th><th>Interacting atoms[b]</th><th>ASA [c]</th><th>BSA [d]</th><th></th><th>Δ^iG [e]</th><th>Conservation[f]</th></tr>
</thead>
<tbody>
<tr><td>E657</td><td>O</td><td>71.8</td><td>1.11</td><td>|</td><td>-0.01</td><td>27.6</td></tr>
<tr><td>N658</td><td>C_α, C, **O**, $O_{\delta1}$, $N_{\delta2}$</td><td>111.4</td><td>28.85</td><td>|||</td><td>-0.28</td><td>25.8</td></tr>
<tr><td>L659</td><td>C_α, O</td><td>20.5</td><td>0.50</td><td>|</td><td>0.01</td><td>98.9</td></tr>
<tr><td>S660</td><td>**O**</td><td>82.6</td><td>8.83</td><td>||</td><td>-0.1</td><td>55.6</td></tr>
<tr><td>E661</td><td>C_α, C_β, C_γ, C_δ, $O_{\varepsilon2}$</td><td>61.4</td><td>29.90</td><td>|||||</td><td>0.29</td><td>100.0</td></tr>
<tr><td>V662</td><td>**N**, O, C_β, $C_{\gamma1}$, $C_{\gamma2}$</td><td>143.9</td><td>64.79</td><td>|||||</td><td>0.49</td><td>67.2</td></tr>
<tr><td>N663</td><td>**N**, C_α, O, C_β, C_γ, **$O_{\delta1}$**, **$N_{\delta2}$**</td><td>97.3</td><td>96.86</td><td>||||||||||</td><td>-0.71</td><td>100.0</td></tr>
<tr><td>P664</td><td>C_g, C_δ</td><td>66.4</td><td>29.97</td><td>|||||</td><td>0.48</td><td>100.0</td></tr>
<tr><td>M665</td><td>C_β, C_γ, S_δ</td><td>48.1</td><td>14.38</td><td>|||</td><td>0.25</td><td>100.0</td></tr>
<tr><td>L666</td><td>C, **O**, C_β, C_γ, $C_{\delta1}$, $C_{\delta2}$</td><td>102.4</td><td>102.23</td><td>||||||||||</td><td>1.17</td><td>80.5</td></tr>
<tr><td>G667</td><td>C_α, C, O</td><td>18.3</td><td>18.30</td><td>||||||||||</td><td>0.29</td><td>100.0</td></tr>
<tr><td>F668</td><td>N, C_α, O, C_β, C_γ, $C_{\delta1}$, $C_{\delta2}$, $C_{\varepsilon1}$, $C_{\varepsilon2}$, C_ζ</td><td>66.1</td><td>49.05</td><td>||||||||</td><td>0.75</td><td>74.2</td></tr>
<tr><td>R672</td><td>C_a, O, C_β, C_γ, C_δ, N_ε, C_ζ, **$N_{\eta1}$**, **$N_{\eta2}$**</td><td>77.0</td><td>75.36</td><td>||||||||||</td><td>-0.6</td><td>100.0</td></tr>
<tr><td>I675</td><td>O, $C_{\gamma2}$, $C_{\delta1}$</td><td>47.0</td><td>46.40</td><td>||||||||||</td><td>0.58</td><td>52.7</td></tr>
<tr><td>S676</td><td>C_α, O, C_β, O_γ</td><td>61.6</td><td>45.12</td><td>||||||||</td><td>0.09</td><td>42.2</td></tr>
<tr><td>Y677</td><td>C_ζ</td><td>95.8</td><td>0.16</td><td>|</td><td>0</td><td>85.8</td></tr>
<tr><td>P678</td><td>C_γ</td><td>43.2</td><td>2.18</td><td>|</td><td>0.03</td><td>99.7</td></tr>
<tr><td>L710</td><td>$C_{\delta2}$</td><td>0.8</td><td>0.17</td><td>|||</td><td>0</td><td>99.9</td></tr>
<tr><td>G712</td><td>C_α</td><td>8.4</td><td>4.16</td><td>|||||</td><td>0.07</td><td>63.7</td></tr>
<tr><td>T713</td><td>C_β, $O_{\gamma1}$, $O_{\gamma2}$</td><td>38.8</td><td>34.94</td><td>||||||||||</td><td>0.47</td><td>29.2</td></tr>
<tr><td>Q715</td><td>C, C_β, C_γ, C_δ, $O_{\varepsilon1}$, $N_{\varepsilon2}$</td><td>102.9</td><td>57.64</td><td>||||||</td><td>0.54</td><td>44.4</td></tr>
<tr><td>E716</td><td>N, C_α, C_γ, C_δ, $O_{\varepsilon1}$, **$O_{\varepsilon2}$**</td><td>31.3</td><td>29.04</td><td>||||||||||</td><td>0.1</td><td>100.0</td></tr>
<tr><td>H719</td><td>C_β, $C_{\delta2}$, **$N_{\eta2}$**</td><td>80.2</td><td>9.59</td><td>|||||</td><td>-0.26</td><td>29.1</td></tr>
<tr><td>I749</td><td>$C_{\gamma1}$, $C_{\gamma2}$, $C_{\delta1}$</td><td>22.8</td><td>2.25</td><td>||||||||||</td><td>0.36</td><td>52.3</td></tr>
<tr><td>P750</td><td>C_β, C_γ, C_δ</td><td>33.0</td><td>2.62</td><td>||||||||||</td><td>0.52</td><td>99.7</td></tr>
<tr><td>R751</td><td>**N**, C_α, C_β, C_γ, C_δ, **N_ε**, C_ζ, **$N_{\eta2}$**</td><td>116.3</td><td>95.03</td><td>|||||||||</td><td>-0.73</td><td>99.9</td></tr>
<tr><td>L754</td><td>O, C_β, $C_{\delta1}$, $C_{\delta2}$</td><td>89.1</td><td>87.55</td><td>||||||||||</td><td>1.2</td><td>81.9</td></tr>
<tr><td>I755</td><td>$C_{\gamma2}$</td><td>29.4</td><td>24.53</td><td>|||||||||</td><td>0.39</td><td>40.3</td></tr>
<tr><td>E758</td><td>C_δ, $O_{\varepsilon1}$, **$O_{\varepsilon2}$**</td><td>80.1</td><td>27.93</td><td>||||</td><td>-0.24</td><td>62.1</td></tr>
<tr><td>Q775</td><td>O, C_γ, $O_{\varepsilon1}$</td><td>5.6</td><td>5.64</td><td>|||||||||||</td><td>-0.01</td><td>100.0</td></tr>
<tr><td>M776</td><td>C_α, O, C_β, C_γ, S_δ, C_ε</td><td>38.7</td><td>38.73</td><td>|||||||||||</td><td>0.34</td><td>57.8</td></tr>
<tr><td>T777</td><td>C_α, **O**, $C_{\gamma2}$</td><td>52.5</td><td>52.48</td><td>||||||||||</td><td>0.14</td><td>88.1</td></tr>
<tr><td>F778</td><td>C_α, C, **O**, C_γ, $C_{\delta2}$, $C_{\varepsilon1}$, $C_{\varepsilon2}$, C_ζ</td><td>63.0</td><td>57.65</td><td>||||||||||</td><td>0.38</td><td>75.4</td></tr>
<tr><td>G779</td><td>N, C_α, **C**</td><td>64.3</td><td>64.25</td><td>||||||||||</td><td>0.23</td><td>99.0</td></tr>
<tr><td>T780</td><td>C_α, C, $C_{\delta1}$, $C_{\varepsilon1}$, O_η</td><td>24.3</td><td>21.30</td><td>|||||||||</td><td>0.16</td><td>38.7</td></tr>
<tr><td>S781</td><td>**N**, C_α, C_β, **O_γ**</td><td>43.0</td><td>42.93</td><td>||||||||||</td><td>0.32</td><td>100.0</td></tr>
<tr><td>R782</td><td>C_γ</td><td>88.6</td><td>0.16</td><td>|</td><td>0</td><td>100.0</td></tr>
<tr><td>D783</td><td>C_β, C_γ, $O_{\delta1}$, **$O_{\delta2}$**</td><td>114.1</td><td>32.05</td><td>|||</td><td>0.15</td><td>100.0</td></tr>
<tr><td>D784</td><td>C, O , C_β, C_γ, **$O_{\delta1}$**, **$O_{\delta2}$**</td><td>76.3</td><td>66.65</td><td>|||||||||</td><td>-0.25</td><td>99.9</td></tr>
<tr><td>V785</td><td>C_α</td><td>12.6</td><td>0.33</td><td>|</td><td>0.01</td><td>80.1</td></tr>
<tr><td>K787</td><td>O, C_β, C_γ, C_δ, C_ε, **N_ζ**</td><td>168.0</td><td>104.09</td><td>|||||||</td><td>-0.77</td><td>54.4</td></tr>
<tr><td>F788</td><td>C_β, C_γ, $C_{\delta1}$, $C_{\delta2}$, $C_{\varepsilon1}$, $C_{\varepsilon2}$, C_ζ</td><td>60.9</td><td>59.74</td><td>||||||||||</td><td>0.96</td><td>99.2</td></tr>
<tr><td>I791</td><td>C_β, $C_{\gamma2}$, $C_{\delta1}$</td><td>109.5</td><td>44.84</td><td>|||||</td><td>0.72</td><td>26.0</td></tr>
<tr><td>T792</td><td>$C_{\delta1}$, $C_{\varepsilon1}$, $C_{\varepsilon2}$, C_ζ, **O_η**</td><td>68.2</td><td>68.24</td><td>|||||||||||</td><td>-0.08</td><td>99.8</td></tr>
<tr><td>E795</td><td>O, C_β, C_δ, **$O_{\varepsilon1}$**, $N_{\varepsilon2}$</td><td>132.2</td><td>34.68</td><td>|||</td><td>-0.34</td><td>59.6</td></tr>
<tr><td>G796</td><td>C, O</td><td>43.4</td><td>10.50</td><td>|||</td><td>-0.06</td><td>57.8</td></tr>
<tr><td>I797</td><td>C_α, O, $C_{\gamma1}$, $C_{\gamma2}$, $C_{\delta1}$</td><td>97.6</td><td>92.70</td><td>||||||||||</td><td>1.11</td><td>79.2</td></tr>
<tr><td>L798</td><td>$C_{\delta2}$</td><td>22.0</td><td>9.37</td><td>|||||</td><td>0.15</td><td>43.1</td></tr>
</tbody>
</table>

Q799	$N_{\varepsilon 2}$	130.5	10.35 |	-0.12	62.7
D807	C_{γ}, **$O_{\delta 1}$**, **$O_{\delta 2}$**	32.2	14.73 |||||	-0.17	99.6
L809	C_{α}, C, O, C_{β}, C_{γ}	112.5	40.42 ||||	0.49	28.3
G810	N, C_{α}, C, O	25.6	25.60 ||||||||||	0.34	100.0
E813	C_{β}, C_{γ}, C_{δ}, $O_{\varepsilon 1}$, $N_{\varepsilon 2}$	106.3	56.61 |||||	-0.57	44.6
L814	C_{γ}, $C_{\delta 1}$, $C_{\delta 2}$	22.7	21.61 ||||||||||	0.35	98.0
M817	S_{δ}, C_{ε}	49.4	20.22 |||||	0.35	44.8
K821	N_{ζ}	66.2	11.32 ||	-0.42	40.7

[a] Interface residues, predicted by PDBePISA.

[b] Residues forming hydrogen bonds and salt bridges are labeled in bold and blue, respectively.

[c] Accessible surface area, in Å^2, predicted by PDBePISA.

[d] Buried surface area, in Å^2, with each vertical bar corresponding to 10% of total solvent-accessible surface area buried, predicted by PDBePISA.

[e] Solvent energy effect, in kcal mol^{-1}, predicted by PDBePISA.

[f] Degree of conservation, from a multiple sequence alignment obtained with MAFFT [4] of 1000 PPDK sequences identified by BLASTp [5] in the NCBI-NR database of non-redundant protein sequences [6].

Table S4: Crystal contacts involving the NBD in PDB ID 5JVJ[a].

Symmetry interface	Interacting atom	HB/ SB	Interacting atom at the NBD	Distance to overlay with alternative NBD conformation [b]
-x-1/2,y-1/2,-z	B-ASP 853 OD1	HB	B-ASN 250 ND2	3.6 (located on loop)
-x-1/2,y-1/2,-z	B-GLN 531 O	HB	B-THR 288 N	0.4
-x-1/2,y-1/2,-z	B-TRP 528 O	HB	B-THR 288 OG1	0.5
-x-1/2,y-1/2,-z	B-THR 362 OG1	HB	B-THR 288 OG1	0.5
-x-1/2,y-1/2,-z	B-ILE 873 O	HB	B-ASP 291 N	0.9
-x-1/2,y-1/2,-z	B-ILE 873 O	HB	B-THR 294 OG1	1.3
-x-1/2,y-1/2,-z	B-ASP 848 O	HB	B-CYS 298 SG	1.6
-x-1/2,y-1/2,-z	B-ASP 848 OD1	HB	B-CYS 298 SG	1.6
-x-1/2,y-1/2,-z	B-LYS 510 N	HB	B-GLU 308 OE1	3.6 (side chain rotated)
-x-1/2,y-1/2,-z	B-LYS 510 NZ	HB	B-GLU 305 OE2	0.8
-x-1/2,y-1/2,-z	B-LYS 510 NZ	SB	B-GLU 305 OE2	0.8
**-x-1/2,y-1/2,-z**	**B-LYS 536 NZ**	**HB**	**B-THR 171 O**	**15.5**
**-x-1/2,y-1/2,-z**	**B-GLN 556 NE2**	**HB**	**B-ASP 172 OD1**	**16.8**
-x-1/2,y-1/2,-z	B-ALA 387 O	HB	B-ARG 315 NH1	5.7 (side chain rotated)
-x-1/2,y-1/2,-z	B-ARG 363 NE	SB	B-GLU 272 OE2	0.8
-x-1/2,y-1/2,-z	B-ARG 363 NH1	SB	B-ASP 291 OD1	0.8
-x+1/2,y-1/2,-z+1	A-SER 390 OG	HB	A-MET 248 O	0.8
**-x+1/2,y-1/2,-z+1**	**A-GLN 511 NE2**	**HB**	**A-GLY 124 O**	**8.4**
**-x+1/2,y-1/2,-z+1**	**A-GLU 290 OE1**	**HB/SB**	**A-LYS 77 NZ**	**11.4**
**-x+1/2,y-1/2,-z+1**	**A-GLU 290 OE2**	**SB**	**A-LYS 77 NZ**	**11.4**
-x+1/2,y-1/2,-z+1	A-SER 390 O	HB	A-ASN 250 N	1.1 (located on loop)
**x,y-1,z**	**A-ARG 476 NH2**	**HB/SB**	**A-ASP 17 OD2**	**8.9**
x,y-1,z	A-ASP 480 OD1	SB	A-LYS 318 NZ	0.6
**x,y-1,z**	**A-ASP 488 OD1**	**HB**	**A-SER 12 N**	**3.0**
**x,y-1,z**	**A-ASP 488 OD1**	**SB**	**A-ARG 11 NE**	**9.0**
**x,y-1,z**	**A-ASP 488 OD2**	**HB/SB**	**A-ARG 11 NE**	**9.0**
**x,y-1,z**	**A-ASP 488 OD2**	**SB**	**A-ARG 11 NH2**	**10.1**
**x,y-1,z**	**B-ASP 650 OD2**	**HB**	**A-LYS 19 N**	**9.8**
**x,y-1,z**	**B-ASP 650 OD2**	**HB**	**A-SER 20 N**	**11.7**
**x,y-1,z**	**B-ASP 650 OD2**	**HB**	**A-SER 20 OG**	**15.3**
**x,y-1,z**	**B-ASP 650 N**	**HB**	**A-SER 20 OG**	**15.3**

[a] As predicted by PDBePISA. Crystal contacts that are unique for an arrangement with chain A (open) and chain B (closed) are shown in bold. Dotted lines separate the crystal contact effecting the NBD of chain B and the crystal contact effecting the NBD of chain A.

[b] Distance of interacting atoms after superposition with the all-atom model of the alternative NBD conformation (see main text for details and Supplementary Fig. S8), in Å.

Supplementary References

1 Krissinel, E. & Henrick, K. Protein interfaces, surfaces and assemblies service PISA at European Bioinformatics Institute. *J. Mol. Biol.* **372**, 774-797 (2007).

2 Minges, A. R. M. *et al.* Structural intermediate and directionality of the swiveling motion of PPDK. *Sci. Rep.* **7**, 45389 (2017).

3 Usami, S., Ohta, S., Komari, T. & Burnell, J. N. Cold stability of pyruvate, orthophosphate dikinase of Flaveria brownii. *Plant Mol. Biol.* **27**, 969-980 (1995).

4 Katoh, K., Misawa, K., Kuma, K.-i. & Miyata, T. MAFFT: a novel method for rapid multiple sequence alignment based on fast Fourier transform. *Nuc. Acids Res.* **30**, 3059-3066 (2002).

5 Altschul, S. F., Gish, W., Miller, W., Myers, E. W. & Lipman, D. J. Basic local alignment search tool. *J. Mol. Biol.* **215**, 403-410 (1990).

6 Coordinators, N. R. Database resources of the National Center for Biotechnology Information. *Nuc. Acids Res.* **41**, D8 (2013).

7 Kabsch, W. & Sander, C. Dictionary of protein secondary structure: pattern recognition of hydrogen□bonded and geometrical features. *Biopolymers* **22**, 2577-2637 (1983).
